# Protocol for Escitalopram and Language Intervention for Subacute Aphasia (ELISA): A randomized, double blind, placebo-controlled trial

**DOI:** 10.1371/journal.pone.0261474

**Published:** 2021-12-23

**Authors:** Melissa D. Stockbridge, Julius Fridriksson, Souvik Sen, Leonardo Bonilha, Argye E. Hillis

**Affiliations:** 1 Department of Neurology, Johns Hopkins University School of Medicine, Baltimore, Maryland, United States of America; 2 Department of Communication Sciences and Disorders, University of South Carolina, Columbia, South Carolina, United States of America; 3 Department of Neurology, University of South Carolina School of Medicine, Columbia, South Carolina, United States of America; 4 Department of Neurology, Medical University of South Carolina, Charleston, South Carolina, United States of America; Cardiff University, UNITED KINGDOM

## Abstract

In this forthcoming multicenter, prospective, randomized, double-blind placebo-controlled trial, we will investigate the augmentative effects of a selective serotonin reuptake inhibitor, escitalopram, on language therapy in individuals with post-stroke aphasia. We hypothesize that, when combined with language therapy, daily escitalopram will result in greater improvement than placebo in an untrained picture naming task (Philadelphia Naming Test short form) administered one week after the end of language therapy. We also will examine whether escitalopram’s effect on language is independent of its effect on depression, varies with lesion location, or is associated with increased functional connectivity within the left hemisphere. Finally, we will examine whether individuals with BDNF met alleles show reduced response to treatment and reduced changes in connectivity. We expect to enroll 88 participants over four years. Participants are given escitalopram or placebo within one week of their stroke for 90 days and receive fifteen 45-minute computer-delivered sessions of language treatment beginning 60 days from the start of drug therapy. Patients then complete a comprehensive assessment of language at one, five, and twenty weeks after the last language therapy session. ELISA is the first randomized, controlled trial evaluating the effect of a selective serotonin reuptake inhibitor on the improvement of language in people with aphasia undergoing language treatment during the acute to subacute post-stroke period.

**Trial registration**: The trial is registered with ClinicalTrials.gov NCT03843463.

## Introduction

Aphasia is a devastating outcome of stroke. The primary intervention is speech and language treatment [SALT; 1, 2]. SALT can be very effective. However, improvement in language through SALT is relatively slow and laborious [3]. While pharmacotherapies that augment plasticity might increase SALT efficiency, few prior studies have evaluated their effect on aphasia rehabilitation [4, 5]. In this project, we will investigate the effects of a selective serotonin reuptake inhibitor (SSRI), escitalopram, on augmenting language therapy, as measured when naming untrained pictures and describing pictures in individuals with aphasia in the acute and subacute post-stroke period (i.e., within three months post stroke).

Escitalopram is a particularly ideal candidate pharmacotherapy as it is a relatively well-tolerated member of its drug class and previously was used in a trial in which it was shown to improve cognitive function [6]. Safety studies reported in the escitalopram label observed only miniscule, sub-clinical changes in QTc (4.5 msec) and found no ECG change when using escitalopram at our intended 10 mg dosing. None of the 625 patients in the escitalopram group had a QT F interval greater than 500 msec or a prolongation >60 msec compared to 0.2% of 527 patients in the placebo group. While there are class-wide concerns regarding an increased risk of depression and suicidality in children, adolescents and young adults taking antidepressants for major depressive disorder (MDD) and other psychiatric disorders, short-term studies did not show an increase in the risk of suicidality with antidepressants compared to placebo in adults beyond age 24 and show a *reduction* in suicidality with antidepressants compared to placebo in adults aged 65 and older. Taken together, these observations support the use of escitalopram as a candidate drug for improving outcomes in the post-stroke adult population.

Escitalopram is a promising adjunct to traditional SALT. We recently found that survivors of left hemisphere stroke who took an SSRI daily for at least three months post-stroke showed better language recovery, compared to those who did not [7]. We first carried out a longitudinal study in which we observed that daily use of SSRIs in the first three months after stroke was associated with better naming recovery, independently of lesion volume, time since stroke, and depression. A large effect size for SSRI use on naming (*d*  =  1.34) was observed. In the confirmatory cross-sectional study, those who took SSRIs continuously for three months post-stroke attained a higher accuracy on object naming than non-users, again with a large effect size [d  =  1.16; 7].

In our ongoing trial examining the effects of transcranial direct-current stimulation (tDCS) versus sham combined with computer-delivered naming therapy on aphasia recovery in subacute stroke, we evaluated the effects of SSRI use (with or without tDCS as we are still masked to treatment condition) on language recovery in the first 22 completers of the study. SSRI use depended on physician’s discretion. We found no significant differences between SSRI users and nonusers in age, education, or any test at baseline. Together, SSRI use, depression score on the Patient Health Questionnaire (PHQ-9), age, and education predicted highest quartile of change in amount of information provided when describing a picture (content units, CU); pseudo r^2^ = 0.62 (p = 0.016). SSRI users were more likely to achieve the highest quartile in CU, compared to nonusers. Although there were no significant differences between SSRI users and nonusers in change on any of the tests (likely due to low power), SSRI users showed higher mean improvements on all tests of language examined compared to nonusers [8]. These data provide pilot evidence that it is possible to recruit, test, and treat participants within three months of stroke onset and preliminary evidence for the investigation of SSRIs in the first three months to augment language therapy for aphasia.

There has been no previous RCT to evaluate the effect of daily SSRI in the first three months after stroke on improvement of language in people undergoing aphasia treatment. It is plausible that SSRIs, which elevate synaptic serotonin, might enhance recovery by augmenting synaptic plasticity. Although several groups have evaluated the effects of pharmacotherapy (plus SALT) on improving language after post-stroke aphasia, most previous studies have been carried out in chronic stroke, usually more than one year after stroke and were relatively small in scale. The few studies of pharmacotherapy for aphasia that have shown substantial positive results have studied medical intervention in the subacute period after stroke [9–11]. Because neuroplasticity and recovery potential are greatest early after stroke, there is reason to believe therapeutic SSRI might be most effective in the acute and early subacute period.

Although there has been relatively little prior investigation of the use of SSRIs in the treatment of post-stroke aphasia, a greater body of work exists that has examined whether SSRIs augment other forms of post-stroke recovery. These trials have led to mixed results. A large, placebo-controlled, randomized clinical trial (RCT) demonstrated that a selective serotonin reuptake inhibitor (SSRI), fluoxetine, results in better motor recovery compared to placebo, if taken daily in the first three months post-stroke [12]. The effect of fluoxetine on motor recovery was independent of effects on depression.

Other RCTs have demonstrated improved cognitive recovery associated with SSRI use after stroke [6, 13]. However, two recent RCTs showed no significant effect of SSRI use on functional outcomes after stroke, measured with the Modified Rankin Scale [mRS; 2, 14]. The mRS is a 6-point scale that is heavily weighted toward motor recovery (e.g., gait). Similar null findings were reported in a recent trial examining the effect of escitalopram on moderate or severe depression in the acute to subacute period, which included numerous measures of functional and motor recovery as secondary outcomes [15]. Nevertheless, a recent meta-analysis of eight trials showed significant effects of SSRI use on functional outcome measured with the NIHSS and the Barthel Index [16, also 17]. Therefore, there remains equipoise regarding the effect of SSRIs on motor recovery. In contrast, the mRS is insensitive to changes in language, other than complete recovery from aphasia (mRS = 0) versus some degree of aphasia at outcome (mRS 1–4, depending on other functional deficits). However, our recent investigations into our own longitudinal data in preparation for the present trial have provided support for examining the use of SSRIs in augmenting language recovery.

Here we describe the protocol for an ongoing Phase II multisite, randomized, double blind, placebo-controlled trial of escitalopram for augmenting language intervention in early subacute stroke. We hypothesize (1.a) that daily escitalopram for 90 days after stroke results in greater improvement (compared to placebo) in naming untrained pictures, as well as greater increase in content of picture description and greater improvement in morphosyntactic production, when combined with SALT. We hypothesize that this effect is independent of escitalopram’s effect on depression (1.b) and most pronounced in individuals with infarcts involving the left superior temporal gyrus and/or arcuate fasciculus compared to individuals without damage to these areas (1.c).

A second aim is to evaluate the mechanisms of language recovery in individuals who receive active medical treatment and those who receive placebo using functional imaging consisting of either resting state functional magnetic resonance imaging (rsfMRI) or functional near-infrared spectroscopy (fNIRS) and genetic testing. We hypothesize that greater improvement in language is associated with increased connectivity within the left hemisphere language network on functional imaging measures in participants who receive escitalopram than in those who receive placebo, independently of improvement in depression (2.a). We will examine whether greater improvement in depression is associated with changes in connectivity in frontolimbic circuits in participants who received escitalopram, but not in those who receive placebo, independently of improvement in language. The goal of this exploratory aim is to determine whether or not we will be able to separate the effects of escitalopram on language and depression, using changes in network connectivity (2.b).

We also hypothesize that the effects are greatest in individuals with val/val allele of brain-derived neurotrophic factor (BDNF; 2.c), consistent with previous studies showing a greater response to treatment and greater neuroplasticity in people with the val/val allele than those with one or more met alleles [18, 19]. Both we [20] and others [21] have shown that language improvement with treatment after stroke is associated with changes in functional connectivity. The presence of a BDNF met allele may hinder the changes in functional connectivity needed for language recovery. In depth connectivity analyses will inform a better understanding the underlying mechanisms by which SSRIs improve language recovery after stroke, contributing to precision, individualized medicine in this population.

## Materials and methods

### Design

**Fig 1:**
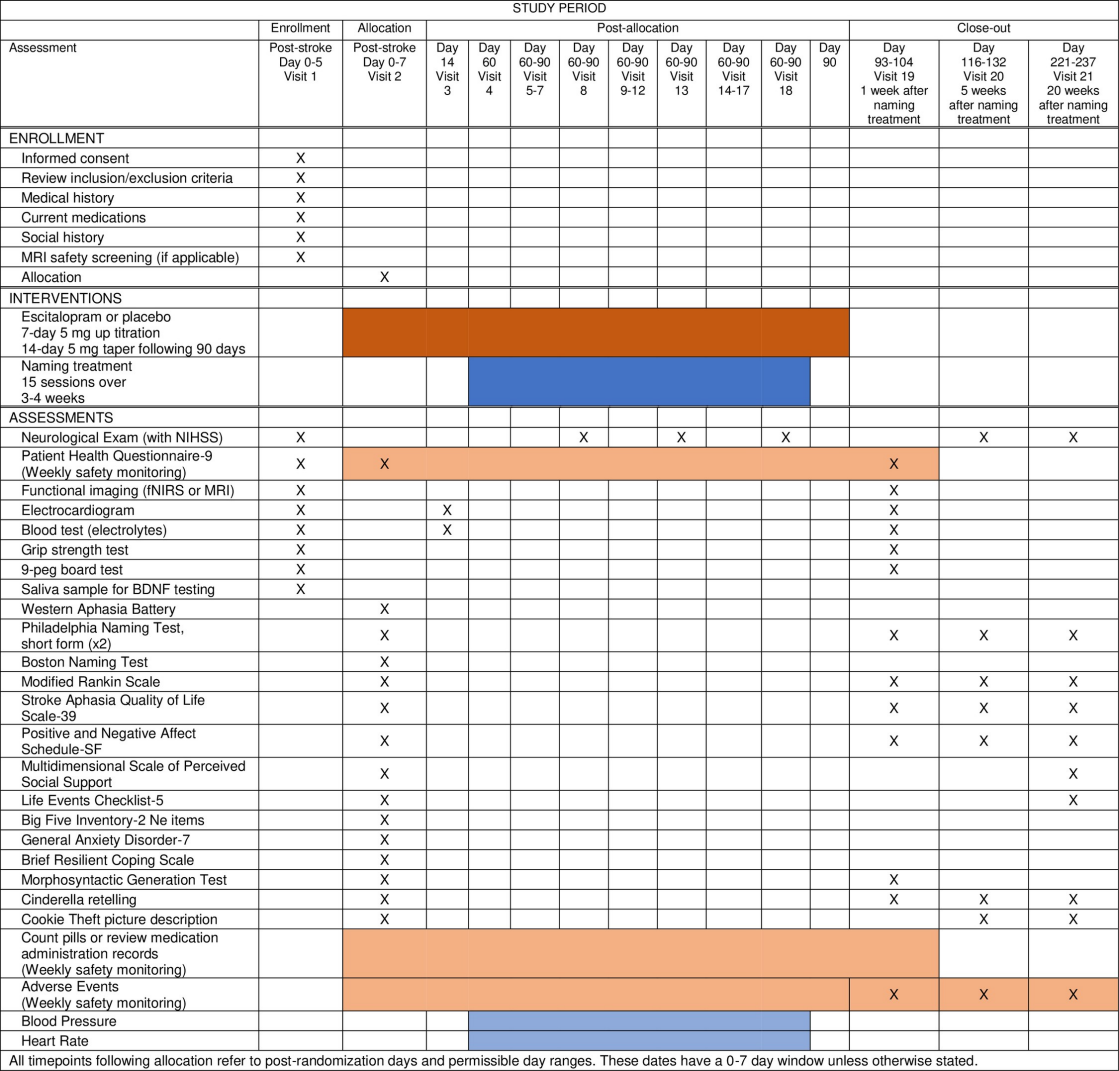
SPIRIT schedule of enrollment, interventions, and assessments.

**Fig 2:**
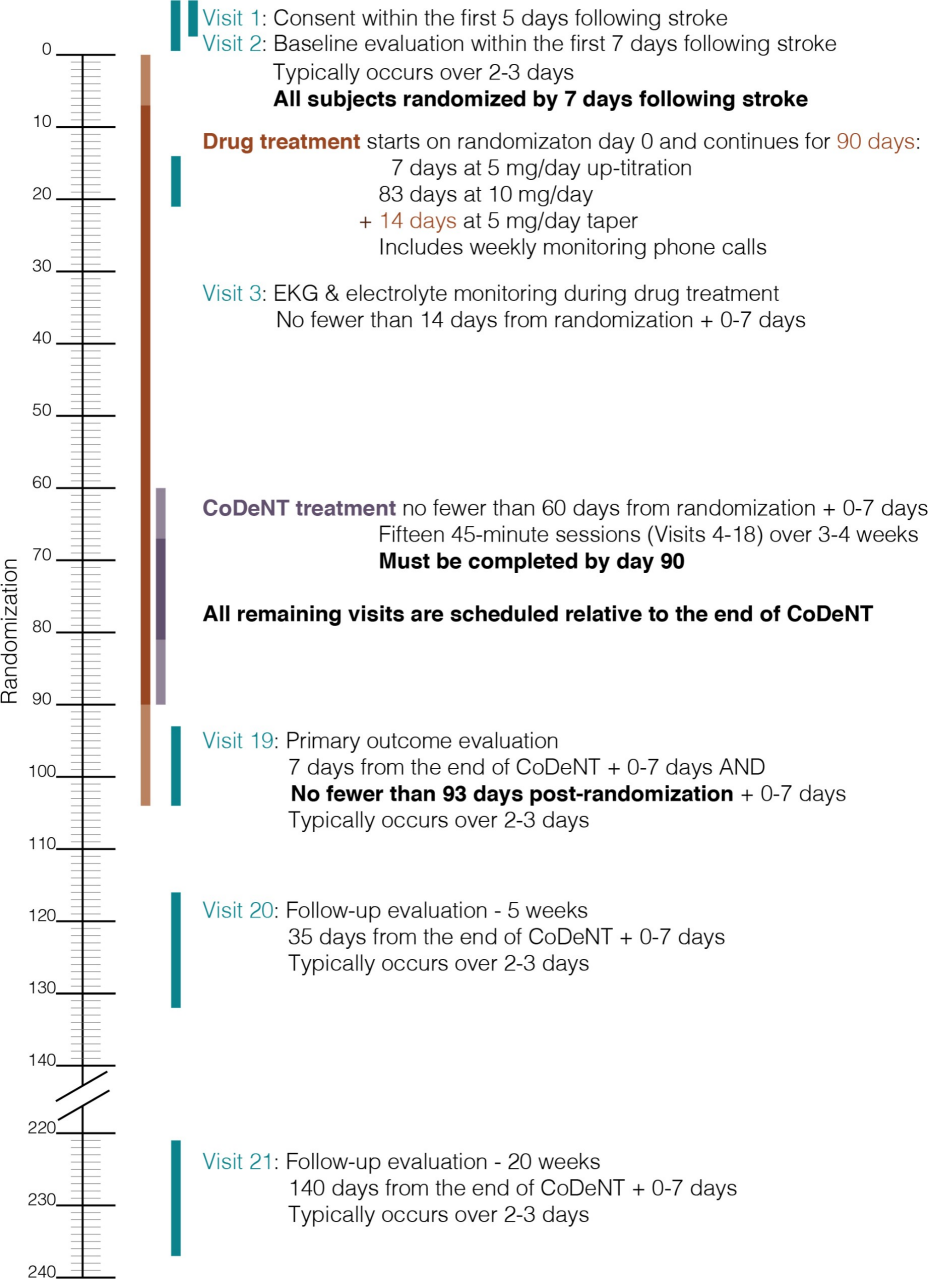
Graphic timeline of key study events.

ELISA is a multicenter, prospective, randomized, double-blind placebo-controlled trial examining the effect of escitalopram daily for 90 days after stroke with concomitant SALT. Aphasia patients with acute ischemic left hemisphere stroke are recruited from Johns Hopkins Medicine, University of South Carolina School of Medicine, and Medical University of South Carolina. All work has been approved by the Johns Hopkins University School of Medicine Institutional Review Board (IRB). Eighty-eight participants are expected to enroll over four years, with at least 56 completing the study on the active or placebo. The SPIRIT schedule of enrollment, interventions, and assessments is included as Fig 1. A graphic timeline is provided in Fig 2. The World Health Organization Trial Registration Data Set compiled by ClinicalTrials.gov (NCT03843463) is reproduced in Table 1 (SPIRIT Item 2b). The SPIRIT checklist is included in S1 File. A full accounting of evaluations and unabridged protocol approved by the IRB is available in S2 File (June 22, 2021; version 1.5) and important protocol modifications will be available from the corresponding author and by viewing the ClinicalTrials.gov study entry. A sample consent form is included as S3 File.

**Table 1:** World Health Organization Trial Registration Data Set.

Data category	Information
Primary registry and trial identifying number	ClinicalTrials.gov NCT03843463
Date of registration in primary registry	February 18, 2019
Secondary identifying numbers	IRB00203667
Source(s) of monetary or material support	National Institute on Deafness and Other Communication Disorders (NIH/NIDCD) P50 DC011739
	Note: This funding source had no role in the design of this study and will not have any role during its execution, analyses, interpretation of the data, or decision to submit results.
Primary sponsor	Johns Hopkins University
Secondary sponsor(s)	None
Contact for public queries	Argye Hillis-Trupe, MD (410) 614–2381 argye@jhmi.edu
Contact for scientific queries	Argye Hillis-Trupe, MD (410) 614–2381 argye@jhmi.edu
Public title	Escitalopram and Language Intervention for Subacute Aphasia (ELISA)
Scientific title	Escitalopram and Language Intervention for Subacute Aphasia (ELISA)
Countries of recruitment	United States of America
Health condition(s) or problem(s) studied	Aphasia, Stroke
Intervention(s)	• Drug: Escitalopram 10mg Escitalopram tablet Other Name: Lexapro • Drug: Placebo Sugar pill manufactured to mimic escitalopram 10 mg tablet Other Name: Placebo (for Escitalopram) • Behavioral: Computer-delivered naming treatment 15 45-minute sessions of computer-delivered naming treatment beginning two months following stroke Other Name: CoDeNT (Computer-delivered naming treatment)
Key inclusion and exclusion criteria	Inclusion Criteria: • Participants must have sustained an acute ischemic left hemisphere stroke. • Participants must be fluent speakers of English by self-report. • Participants must be capable of giving informed consent or indicating a legally authorized representative to provide informed consent. • Participants must be age 18 or older. • Participants must be within 5 days of onset of stroke. • Participants must be pre-morbidly right-handed by self-report. • Participants must have an aphasia diagnosis as confirmed by the Western Aphasia Battery-Revised (Aphasia Quotient < 93.8). • Exclusion Criteria: • Previous neurological disease affecting the brain including previous symptomatic stroke • Diagnosis of schizophrenia, autism, or other psychiatric or neurological condition that affects naming/language • A history of additional risk factors for torsades de pointes (TdP; e.g., heart failure, hypokalemia, family history of Long QT Syndrome) • Current severe depression, defined as a score of > 15 on the Patient Health Questionnaire (PHQ-9) • Uncorrected visual loss or hearing loss by self-report • Use of any medication approved by the FDA for treatment of depression at the time of stroke onset • Concomitant use of any monoamine oxidase inhibitors (MAOIs) or pimozide, or other drugs that prolong the QT/QTc interval, triptans (and other 5-Hydroxytryptamine Receptor Agonists), or other contraindications to escitalopram that may be identified. • A QTc greater than 450 milliseconds on electrocardiogram or evidence of hyponatremia (Na < 130) at baseline • Pregnancy at the time of stroke or planning to become pregnant during the study term.
Study type	Interventional
	Allocation: Randomized
	Intervention model: Parallel Assignment
	Masking: Triple (Participant, Care Provider, Investigator
	Primary purpose: Treatment
	Phase II
Date of first enrollment	July 18, 2021
Target sample size	60
Recruitment status	Recruiting
Primary outcome(s)	Change in Philadelphia Naming Test short form accuracy score [ Time Frame: Baseline, 1 week after computer-delivered naming treatment ]
Key secondary outcomes	2. Language production as assessed by lexical features of discourse in "Cookie Theft" picture description Lexical features, meaning carrying units of language (morphemes), will be counted for each Cookie Theft picture description. There is no maximum number of meaning carrying units, but norms are available to assist in the interpretation of this performance. [Time Frame: Baseline, 5 weeks after computer-delivered naming treatment] 3. Language production as assessed by content units included in picture description of "Cookie Theft" Content units are based on a standard scoring template of commonly identified concepts (nouns and verbs) in the left and right regions of the "Cookie Theft" picture. Participants either include or fail to include 30 concepts on the left side of the picture and 23 concepts on the right side of the picture. A ratio of included left content units to included right content units then can be calculated and interpreted as a measure of hemispatial attention. [Time Frame: Baseline, 5 weeks after computer-delivered naming treatment] 4. Language production as assessed by rate of syllables per content unit produced in "Cookie Theft" picture description Syllables included in the picture description are counted. Content units are based on a standard scoring template of commonly identified concepts (nouns and verbs) in the left and right regions of the "Cookie Theft" picture. Participants either include or fail to include 30 concepts on the left side of the picture and 23 concepts on the right side of the picture. The average rate of syllables per content unit produced can then be calculated and interpreted as a measure of efficiency in producing relevant information in the task. [Time Frame: Baseline, 5 weeks after computer-delivered naming treatment] 5. Depression as assessed by Patient Health Questionnaire (PHQ-9) 9 item scale scored 0–3 for each item. PHQ-9 scores of 5, 10, 15, and 20 represent mild, moderate, moderately severe, and severe depression. PHQ-9 >15 or suicidal ideation suggest depression sufficient for exclusion or removal from study. [Time Frame: Baseline, 1 week after computer-delivered naming treatment] 6. Language production as assessed by Morphosyntactic Generation (MorGen) Test 60 item assessment of word morphology (e.g., plurals, possessives) and modifiers (e.g., number, size, color). Each item is scored based on produced accurate descriptors of an image relative to a second reference image (e.g., patients see two trees, one larger than the other, and the phrase "little tree" is elicited). Patients are scored for objects correctly named (nouns) out of 60, instances of correct use of plural marker out of 31, instances of correct use of numbers out of 8, instances of correct modifiers denoting size out of 16, instances of correct modifiers denoting color out of 19, instances of correct modifiers denoting possessive markers out of 17, and instances of correctly named possessing individuals (proper names provided on screen) out of 17. These scores can then be interpreted separately or averaged to interpret a broad morphosyntactic accuracy score. [Time Frame: Baseline, 1 week after computer-delivered naming treatment] 7. Stroke severity as assessed by NIH Stroke Scale (NIHSS) The NIHSS is a 15-item neurologic examination stroke scale used to evaluate the effect of acute cerebral infarction on the levels of consciousness, language, neglect, visual-field loss, extraocular movement, motor strength, ataxia, dysarthria, and sensory loss. A trained observer rates the patient’s ability to answer questions and perform activities. Ratings for each item are scored with 3 to 5 grades with 0 as normal, and there is an allowance for untestable items. [Time Frame: Baseline, during computer-delivered naming treatment, 5 weeks after computer-delivered naming treatment, 20 weeks after computer-delivered naming treatment] 8. Post-stroke level of disability as assessed by modified Rankin Scale (mRS) The mRS is a 6-level scale from "0-No symptoms" to "6-dead" used to evaluate the degree of disability in patients who have suffered a stroke. [Time Frame: Baseline, 1 week after computer-delivered naming treatment] 9. Stroke paresis severity as assessed by right hand strength Right hand strength assessment by dynamometer [Time Frame: Baseline, 1 week after computer-delivered naming treatment] 10. Stroke paresis severity as assessed by right hand dexterity Right hand dexterity assessment by 9 peg board test [Time Frame: Baseline, 1 week after computer-delivered naming treatment] 11. Change in new vocabulary items as assessed by lexical diversity included in story retelling of "Cinderella" Change in new vocabulary items will be counted for each noun, verb, and adjective in the Cinderella retelling. There is no maximum measure of lexical diversity, but norms are available to assist in the interpretation of this performance. [Time Frame: Baseline, 1 week after computer-delivered naming treatment] 12. Change in incidence of new vocabulary items as assessed by lexical diversity included in story retelling of "Cinderella" Change in incidence of each new item will be counted for each noun, verb, and adjective in the Cinderella retelling. There is no maximum measure of lexical diversity, but norms are available to assist in the interpretation of this performance. [Time Frame: Baseline, 1 week after computer-delivered naming treatment] 13. Change in language production as assessed by speech errors produced during the story retelling of "Cinderella" Change in number of errors will be counted after each retelling is recorded [Time Frame: Baseline, 1 week after computer-delivered naming treatment] 14. Change in Language production as assessed by speech pauses produced during the story retelling of "Cinderella" Change in pauses will be counted after each retelling is recorded [Time Frame: Baseline, 1 week after computer-delivered naming treatment]

### Patient population–inclusion and exclusion criteria

Participants must be within five days of ischemic left-hemisphere stroke and diagnosed with post-stroke aphasia using the Western Aphasia Battery-Revised (WAB-R; 4). They also must be right-handed and English-speaking with no history of major psychiatric or neurological disorder affecting the brain, antidepressant use at time of stroke, depression (PHQ-9 score ≥15), or risk factors for study drug (including QTc interval > 450 msec or sodium < 130). Patients with current severe depression are excluded.

### Inclusion criteria

1. Participants must have sustained an acute ischemic left hemisphere stroke.
2. Participants must be fluent speakers of English by self-report.
3. Informed consent must be obtained from the participant or legally authorized representative.
4. Participants must be age 18 or older.
5. Participants must be pre-morbidly right-handed by self-report.
6. Participants must be within 5 days of onset of stroke at the time of consent.
7. Participants must have an aphasia diagnosis as confirmed by the Western Aphasia Battery-Revised (Aphasia Quotient, AQ < 93.8).

### Exclusion criteria

1. Previous neurological disease affecting the brain including previous symptomatic stroke
2. Diagnosis of schizophrenia, autism, or other psychiatric or neurological condition that affects naming/language
3. A history of additional risk factors for torsades de pointes (TdP; e.g., heart failure, hypokalemia, family history of Long QT Syndrome)
4. Current severe depression, defined as a score of > 15 on the Patient Health Questionnaire (PHQ-9) or endorsing suicidality (PHQ-9 question 9)
5. Uncorrected visual loss or hearing loss by self-report
6. Use of any medication approved by the FDA for treatment of depression at the time of stroke onset
7. Concomitant use of any monoamine oxidase inhibitors (MAOIs), pimozide and other drugs that prolong the QT/QTc interval, triptans (and other 5-Hydroxytryptamine Receptor Agonists), or other contraindications to escitalopram that may be identified. See 5.3.3. Prohibited interventions for full details.
8. A QTc greater than 450 milliseconds on electrocardiogram.
9. Evidence of hyponatremia (Na < 130) at baseline.
10. Pregnancy at the time of stroke or planning to become pregnant during the study term.

### Informed consent

A dated and signed consent form will be obtained from each participant. For participants who cannot consent for themselves, such as those with a legal guardian (e.g., person with power of attorney), this individual must sign the consent form. The consent form will describe the purpose of the study, the procedures to be followed, and the risks and benefits of participation. A copy will be given to each participant or legal guardian and this fact will be documented in the participant’s record.

Participants may elect not to participate in the genetic testing, MRI, or fNIRS procedures of the study and still complete the drug trial. Participants will then be assigned a temporary identification number for the purposes of initial screening.

All research staff authorized to obtain informed consent will have completed the Miami CITI course in the Responsible Conduct of Research and Protection of Human Subjects prior to their involvement with the study. Furthermore, they will be oriented to the study and trained by the study principle and co-investigators who have all had extensive training and experience in the ethical and practical aspects of informed consent procedures.

### Participant confidentiality

Participation in this study should not put participants in any legal risk, even in the case of a breach of confidentiality. We will undertake every effort to keep the information in the study confidential. Participants will be assigned a code number in order to keep the information confidential. The networks on which the information will be stored are password protected. Everybody involved in the study will have completed the appropriate HIPAA training and are fully aware of confidentiality issues. No names will be included in any publications resulting from this work

Any data, specimens, forms, reports, video recordings, and other records that leave the site will be identified only by a participant identification number to maintain confidentiality. All records will be kept in a locked file cabinet. All computer entry and networking programs will be done using participant identification numbers only. Information will not be released without written permission of the participant, except as necessary for monitoring by IRB, the FDA, the NIDCD, and the OHRP.

### Randomization

All eligible patients receive comprehensive language evaluations of prior to randomization. Randomization is 1:1 (escitalopram or placebo), controlling for severity on the WAB-R. All randomized participants will be included in the intent-to-treat analysis.

The randomization will take place centrally via the trial website. The computer program developed at the Medical University of South Carolina Data Coordination Unit (DCU) makes the treatment assignment based on the current status of treatment group distribution within each stratum as well as overall balance of treatment assignment and implements a “real-time” randomization procedure.

The center staff enters the baseline WAB-R Aphasia Quotient (aphasia severity) and eligibility information of a subject prior to enrollment. If the subject’s eligibility status is confirmed, the computer program on the WebDCU^TM^ server will evaluate the treatment arm distribution and generate a blinded treatment assignment based on the randomization scheme. The research team member randomizing the participant will not see the treatment assignment, only a numeric participant number. The unblinded list of randomization codes and treatment assignments will be generated by the DCU and communicated to the research pharmacies. There are no sample size goals for each stratum.

### Treatment

Drug therapy will be started within the first seven days after stroke and participants receive escitalopram or placebo daily for 90 days. Escitalopram is a well-tolerated SSRI that has been shown to be effective in treatment of depression and anxiety at a dosage of 10 mg per day. The FLAME trial found that fluoxetine 20 mg per day for three months after stroke onset was associated with greater motor recovery compared to placebo [12] and the same dosage was used in other large clinical trials in post-stroke recovery [14]. The equivalent dose of escitalopram is 9 mg per day [22]. Escitalopram is commercially available at a dose of 10 mg, and this is the recommended therapeutic dosage for individuals 65 and older. Thus, we will use 10 mg as the study dose. This dose was also used in the positive RCT of escitalopram to improve cognitive function post-stroke [6].

Escitalopram has been associated with spontaneous reports of adverse events occurring upon discontinuation, particularly when abrupt. The most common side effects are drowsiness, nausea, insomnia, dry mouth, constipation, which are also common with placebo. Participants will be up-titrated onto the medication by taking 5 mg escitalopram (or placebo) for the first week of treatment and tapered off the medication by taking 5 mg escitalopram (or placebo) for 2 weeks after the 90 days of treatment. All participants are monitored daily during hospitalization and weekly after discharge until the end of trial for signs of depression, adverse events, and compliance.

Intervention for a participant will be discontinued if any of the following criteria are met:

- The participant requests removal from the study
- The participant exhibits high suicidality/severe depression (PHQ-9 > 15)
- The participant exhibits hyponatremia (Na < 130)
- The participant exhibits a QTc greater than 450 milliseconds
- The participant is diagnosed with schizophrenia or another psychiatric or neurological condition that affects naming/language (self-report)
- The participant is diagnosed with a psychiatric condition requiring interventions prohibited while on escitalopram (e.g., severe depression, bipolar disorder, by self-report).
- The participant experiences sudden visual or hearing loss (self-report)
- The participant becomes pregnant during the drug treatment (self-report)
- The participant starts a medication that is contraindicated (self-report) For a full list of contraindicated medications, see the full protocol in the supplement, section 5.3.3.

As long as it is not medically inadvisable, participants whose treatment is discontinued during the drug treatment term will complete a 5 mg taper off of escitalopram for 2 weeks in order to mitigate the risk for side-effects due to discontinuation of the drug. No additional evaluations will be conducted following discontinuation. The study may be discontinued at any time by the IRB, the NIDCD, the OHRP, the FDA, or other government agencies as part of their duties to ensure that research participants are protected. The study may be discontinued if the study is closed by Johns Hopkins School of Medicine or by the overseeing Center for the Study of Aphasia Recovery (C-STAR).

Sixty days after randomization, SALT is initiated with fifteen 45-minute online computer-delivered naming therapy sessions. Participants may also receive routine SALT throughout the study, which will be documented. We selected this naming treatment and timing to compare results of escitalopram plus SALT to the effects of tDCS plus SALT in subacute stroke in an ongoing trial (NCT02674490).

### Primary outcome

The primary outcome is defined as the change in number of correctly named items on the 30-item Philadelphia Naming Test short form [PNT; 23] from baseline to one week after computer-delivered naming therapy.

### Secondary outcomes

Several secondary analyses will be conducted on the linguistic, cognitive, personality and temperament, neuroimaging, and genetic data collected in addition to the PNT prior to randomization and 1-, 5-, and 20-weeks after the computer-delivered naming therapy. Secondary objectives will test hypotheses related to the relationship between language improvement and (1.b.) antidepressant effects, (1.c.) lesion location, (2.a.) functional connectivity in the left hemisphere language network, (2.b.) connectivity in the frontolimbic circuits, and (2.c.) met alleles of BDNF.

We will evaluate the proposed mechanism of SSRI on neural mechanisms underlying the effects of an SSRI on augmenting language recovery using longitudinal rsfMRI and genetic testing. To supplement the fMRI data, we will also collect task-based and task-free (“resting”) fNIRS data. The fNIRS protocol is expected to take up to 60 minutes. fNIRS is a safe, non-invasive, and flexible modality for brain imaging. During an fNIRS experiment, an array of light sources and detectors affixed to a cap is placed on the scalp, and the measures from these different channels allow the reconstruction of an image of the hemodynamic response. fNIRS has emerged as a complementary technology to other brain imaging and monitoring modalities. Previous research [24, 25] in healthy populations has shown that the hemodynamic response captured in fNIRS is similar to that measured in fMRI. Collecting either fMRI or fNIRS data at each time point in the present study will allow us to recruit participants for whom MRI is contraindicated or logistically prohibitive and will provide greater flexibility to complete assessments outside of the hospital. Similar to task-free fMRI, “resting” fNIRS will allow us to determine intrinsic functional connectivity between regions of the brain at each stage of stroke recovery. In the event of a pandemic or other event that prevents participants from having MRI, we will be able to use fNIRS to test hypotheses about changes in connectivity associated with treatment, as the fNIRS equipment is not only highly portable, but can also be disinfected relatively easily after each use.

### Blinding

The study is to be conducted in a double-blind manner. The subjects, the site investigators, and the clinical staff involved in this study will not know the treatment assignment. The statistical team at the Statistical and Data Management Center will be unblinded. The study statistician will provide a sealed envelope with the treatment group identifiers to the data safety monitoring board (DSMB). This envelope would only be opened by the DSMB if they require unblinding or at the end of the study.

### Data collection and quality assurance

A blinded speech-language pathologist (SLP) to be named at each site will perform baseline and outcome assessments (described in full in the unabridged protocol found in the Supplement). Codes without any protected health information will be used for each participant. Demographic and assessment data will be recorded directly on the assessment forms, and this will be considered source data. The forms will be kept in locked cabinets and secured servers and will be entered into WebDCU^TM^. After assessments are scored by raters, these data are entered, monitored and analyzed in WebDCU^TM^ at the Medical University of South Carolina.

The Clinical Core (authors 2–5) is responsible for training all personnel who have contact with participants and/or friends and family members to ensure that all exchanges are ethical, respectful, and employ successful communication strategies. This includes training for communication during initial recruitment and participant identification, for the informed consent process, and for participant retention. It also includes training of assessment administration procedures and treatment procedures. This training helps to reduce variance introduced by clinicians communicating or carrying out procedures with different styles than others. Training of personnel in this manner has rarely been reported in the aphasia treatment literature.

The Clinical Core ensures that all study personnel having any contact with participants and/or the friends and family members of the participants have a basic understanding of the communication needs and obstacles for this population, and also some understanding of their experience. While most study personnel have had a background with adult neurogenic communication disorders, standardizing this baseline training for all personnel, from those who might only be escorting them to the elevator to those who spend hours with them in treatment sessions, ensures that no erroneous assumptions about qualifications are made. This baseline training comprises many components that are already implemented by the Clinical Core of C-STAR, with positive feedback and results. The Clinical Core continues to facilitate the training and maintain compliance logs.

First, all study personnel complete human subjects training (CITI) to understand ethical treatment of human research participants, which is essential training even in the earliest stages of a study (e.g., to avoid coercion and/or bias during advertising and recruitment efforts). Second, all personnel independently read two articles by the Hilari research group discussing the impact of aphasia. Third, all personnel independently view four videos available via AphasiaBank (http://talkbank.org/AphasiaBank/; participants consented to be videotaped by AphasiaBank) during which persons with aphasia discuss their stroke story. Two videotaped persons have nonfluent aphasia (1 high, 1 low severity) and two have fluent aphasia (1 high, 1 low severity). Finally, the Clinical Core leads small group sessions. The stated goals of the program are: (1) To give participants an opportunity to experience what it is like to be communicatively impaired; (2) To encourage participants to discuss their emotional responses to being treated as an impaired individual; and (3) To instruct participants about the different modalities that may be impaired as a result of stroke or head injury. This session will provide opportunities for personnel to participate in simulations of communicating with aphasia through receptive language, expressive language, reading, and writing stations.

Borrowing from treatment fidelity guidelines, the Clinical Core will coordinate assessor and rater training, assessment delivery, and reliability of raters. Assessors are speech-language pathologists who already have a basic understanding of the project (through baseline and informed consent training, some of whom already have received the detailed training described below, facilitated by the current Clinical Core. New assessors will complete the same training. Illustrating the relationships between the assessments the clinicians are giving and the specific project goals emphasize the importance of the assessment process and adherence to prescribed procedures. This development of clinician “meta-competence” or “buy-in” is thought to be important in treatment fidelity and is intuitive in assessment as well–understanding the “why” facilitates investment in the “how”, or adherence to procedures. In addition, highlighting the opportunities most susceptible to drift or contamination serves to bring the information to the forefront so that it can be actively avoided. Finally, clinician attrition is likely to be observed less in clinicians who are invested in the project goals and feel as if their contribution is important.

The Clinical Core will facilitate the following assessment training procedures: (1) independent reading of the assessment manual; (2) video observation of expert administration of each test, administered to persons with varying types and severity of aphasia; (3) a small group training session that includes manual review, highlighting of similarities and differences between administration procedures via discussion and video observation, and supervised role-play with feedback; (4) at project initiation, three supervised assessment sessions with expert feedback; and (5) throughout study, yearly “booster” small group training and 1–2 supervised assessment sessions with expert feedback. These training procedures guard against clinician-to-clinician variability, drift, and contamination. Both new and current assessors will receive specialized training in administration of the Morphosyntactic Generation (MorGen) test. To continue training, SLPs meet monthly (or more often) to discuss questions about protocols. They also participate in online webinars and other professional development activities related to aphasia and apraxia.

No additional auditing of trial conduct is planned.

### Sample size estimates

Eighty-eight participants are expected to enroll in this study. We expect at least 56 will complete the study on the study drug. However, all participants randomized will be included in the intent-to-treat analysis. A final sample size of 28 per group has 90% power to detect an effect size of d = 0.8 using a *t*-test with α = 0.05, one-tailed. The planned sample size was inflated from N = 56 to N = 88 to account for up to 20% attrition or non-compliance, using an inflation factor of R = 1/(1–0.20)^2^. Effect sizes >1 in naming improvements amongst post-stroke patients who took SSRIs for three months SSRIs have been reported [7]; however, we have conservatively powered this study for a smaller effect.

We predict no difficulty recruiting at least 22 people with aphasia due to acute stroke each year. The PI has recruited an average of 13 people with aphasia due to acute left hemisphere ischemic stroke each year in her ongoing treatment study with tDCS at Johns Hopkins Hospital and Johns Hopkins Bayview Medical Center, with similar inclusion and exclusion criteria. We anticipate having more difficulty enrolling in this trial, because of the exclusion criteria of moderately-severe depression or use of SSRIs at the time of stroke. We also plan for a relatively high dropout rate/crossovers, because of the risk of developing moderate-severe depression or other events for which stroke patients are at risk (e.g. recurrent stroke) and a small risk of developing prolonged QTc interval on electrocardiogram on escitalopram.

### Statistical analyses

To the primary outcome measure, we will compare change in accuracy of naming untrained items between groups in a *t*-test. Missing data will be imputed using a multiple imputation approach assuming a monotone missing mechanism and missing is at random (MAR).

Secondary analyses will examine if any effects of escitalopram vary across participants with different characteristics such as age, education, lesion location and volume using regression models as follows:

1.b. Fit a linear regression of the primary outcome measure, which includes main effects for baseline depression, treatment group, and their interaction. We anticipate that the effect of escitalopram (versus placebo) will not vary by depression subgroup.
1.c. Fit a linear regression of the primary outcome measure, which includes main effects for lesion location, treatment group, and their interaction. We anticipate that the effect of escitalopram will be greater in those with superior temporal gyrus/arcuate fasciculus infarcts.
2.a. Fit a linear regression of the primary outcome measure, which includes main effects for mean connectivity across nodes in the language network, treatment group, and their interaction, adjusting for baseline depression.
2.b. Fit a linear regression of change in PHQ-9 score, which includes main effects for mean connectivity across nodes in the frontolimbic network, treatment group, and their interaction.
2.c. Fit a linear regression of the primary outcome measure, which includes main effects for BDNF mutations, treatment group, and their interaction. We will fit a similar model using connectivity instead of naming accuracy as the dependent variable.

Since these are secondary, exploratory analyses in a small sample, a significance level of 0.10 will be used to retain main effects or interaction terms with treatment in the final reported model.

No formal interim analyses are planned. If the DSMB determines that there is a safety concern, or the PI or IRB identifies a safety concern, the study will be stopped.

### Data monitoring body

The study biostatistician will generate closed and open reports semi-annually or more frequently, as determined by the DSMB, which provide statistics on enrollment, participant status, safety data, and data quality information. The DSMB trial consists of accomplished scientists in Neurology, Epidemiology, Psychiatry, and Physical Medicine & Rehabilitation from external institutions and will monitor safety at least semi-annually and decide if the study should continue or be terminated early. DSMB members include Steven C. Cramer, MD, FAAN, FAHA (David Geffen School of Medicine at UCLA, California Rehabilitation Institute), Robert G. Robinson, MD (Roy J. and Lucille A. Carver College of Medicine, University of Iowa), Steven L. Small, PhD, MD (University of Texas at Dallas), and Maureen G. Maguire, PhD (Perelman School of Medicine, University of Pennsylvania).

### Specification of safety parameters

The participant may stop testing or the intervention any time. There will be emergency personnel and equipment on hand for safety during in-person visits. QTc will be evaluated with electrocardiogram (ECG), reviewed by the consulting cardiologist at JHMI, before starting escitalopram (Visit 1), during the drug treatment period (Visit 3), and approximately one week following the end of CoDeNT (Visit 19). ECGs obtained at each site will be scanned and sent to the consulting cardiologist to securely to review. If an unexpected finding is identified, the consulting cardiologist will ask for permission to contact the primary care physician and will arrange a prompt evaluation by a cardiologist or other appropriate physician. Sodium and other electrolytes will be evaluated at the same visits. If participants have a prolonged QTc or significant hyponatremia (Na<130), they will be removed from the study drug treatment but will continue to be followed through the end of the study term.

If an unexpected finding is identified on MRI, the study physician (Dr. Hillis or the site PI) will explain the finding and will ask permission to contact the primary care physician and will arrange prompt evaluation by a neurologist, neurosurgeon, or other appropriate physician.

An adverse event (AE) is defined as any unfavorable and unintended diagnosis, symptom, sign (including an abnormal laboratory finding), syndrome or disease which either occurs during the study, having been absent at baseline, or if present at baseline, appears to worsen. A serious adverse event (SAE) is any untoward medical occurrence that results in death, is life threatening, requires inpatient hospitalization or prolongation of existing hospitalization, results in persistent or significant disability/incapacity, or is a congenital anomaly. Electrocardiograms and blood tests will be collected to assess safety. Thresholds for exclusion at the pre-treatment evaluation or removal from the drug treatment at the mid-treatment visit are:

- Hyponatremia (Na < 130)
- QTc greater than 450 milliseconds

No AE will be specifically solicited beyond the evaluations before and immediately following treatment. Unsolicited events will be monitored during all visits as reported by participants and captured in the AE case report form.

Adverse events will be monitored during the entire visit by the study team. The families will be given telephone numbers of study team as well. The site PI and the DSMB will be notified immediately if any serious adverse events are reported. DSMB members will review SAEs within 24 hours of when the PI or other study team member becomes aware of the SAE and will determine if the SAE is related to the study, and what actions (if any) are required in response to the SAE. If a significant safety concern arises, participants may be unblinded in order to address it. Adverse events will be monitored until they are resolved or clearly determined to be due to a subject’s stable or chronic condition or intercurrent illness. Medical care will be provided, as defined in the informed consent, for any adverse event related to trial participation. Appropriate medical care will include initiating transport to the Emergency Department for evaluation when necessary. All adverse events, regardless of intensity or causality, will are to be recorded in the study documentation and reported to the JHU IRB and DSMB. Any serious adverse events will be reported to the IRB and the DSMB within 24 hours.

All adverse experiences will be summarized in terms of frequency, severity and relatedness to the study treatment using the MedDRA code. All subjects who received escitalopram will be included in the safety analysis. At the end of the study, the cumulative incidences of adverse events are compared between the two treatment groups using Fisher’s exact test at the two-sided alpha level of 0.05.

The study biostatistician will generate closed and open DSMB reports semi-annually or more frequently, as determined by the DSMB. Each DSMB report provides cumulative summary statistics on enrollment; subject status in the study (e.g., number completed study, dropouts, etc.); baseline characteristics; safety data, including AEs and SAEs; and data quality information. The statistics for the closed DSMB Reports are provided by treatment group displayed as A or B. The open report contains aggregated statistics only, i.e., not by treatment group.

## Summary and concluding remarks

The effect of acute and early subacute SSRI with concomitant SALT on language recovery remains incompletely understood. It is our hope that these findings will support more individualized, effective, and efficient treatment recommendations for individuals who experience language deficits following stroke. Regardless of outcome, results will be disseminated in peer-reviewed journals and meaningfully contribute to the growing body of literature on the topic of pharmacological therapy to facilitate post-stroke rehabilitation, which historically has focused on motor and cognitive outcomes with mixed results.

If this trial shows benefits of SSRIs on post-stroke language recovery, a next step would be to conduct a full Phase III clinical trial and examine whether this is a suitable indication for this drug in the label. This would be supported by complementary investigations to better understand the underlying mechanism of action, whether directly associated with serotonergic changes to plasticity or mediated by the SSRI’s impact on affect. A subsequent investigation would examine whether the combination of tDCS and SSRIs behave synergistically in the treatment of post-stroke aphasia.

## Supporting information

S1 File SPIRIT checklist. (DOCX)

S2 File Protocol. (DOCX)

S3 File Consent form. (PDF)
