# Supplementary material for: Protocol for Escitalopram and Language Intervention for Subacute Aphasia (ELISA): A randomized, double blind, placebo-controlled trial
Source: PLoS One. 2021 Dec 23;16(12):e0261474. doi: 10.1371/journal.pone.0261474 (PMC8699636; doi:10.1371/journal.pone.0261474)
Supplement: S2 File — (DOCX) [file pone.0261474.s002.docx]

# Escitalopram & Language Intervention for Subacute Aphasia

**(ELISA)**

Principal Investigator: Argye Hillis Trupe, M.D.

Professor of Neurology

Executive Vice Chair, Neurology

Director, Cerebrovascular Division

Johns Hopkins University School of Medicine

Supported by: National Institute on Deafness and

Other Communication Disorders (NIH/NIDCD)

P50 DC011739

Study Intervention Provided by: Each site’s investigational pharmacy, ordered from the site’s pharmaceutical catalogue (active generic escitalopram) or Temple University, Dr. David Lebo (placebo).

# Tool Revision History

Version Number: 1.1

Version Date: 9/27/2020

Summary of Revisions Made: Original document

Version Number: 1.2

Version Date: 1/25/2021

Summary of Revisions Made: Clarifications and changes made to bring protocol into compliance with relevant FDA guidances for clinical trials.

Version Number: 1.4

Version Date: 4/27/21

Summary of Revisions Made: Naming screening removed from baseline testing. No other substantive procedural changes. Minor changes to wording requested by the clinical trial data management team have been honored.

Version Number: 1.5

Version Date: 6/22/2021

Summary of Revisions Made: Inclusion of additional manufacturers of escitalopram 5mg tablets.

# TABLE OF CONTENTS

Page

[Escitalopram & Language Intervention for Subacute Aphasia 1](#_Toc62220549)

[Tool Revision History 2](#_Toc62220550)

[TABLE OF CONTENTS 3](#_Toc62220551)

[STUDY TEAM ROSTER 6](#_Toc62220552)

[PRÉCIS 7](#_Toc62220553)

[1. STUDY OBJECTIVES 9](#_Toc62220554)

[1.1 Primary Objective 9](#_Toc62220555)

[1.2 Secondary Objectives 9](#_Toc62220556)

[2. BACKGROUND AND RATIONALE 10](#_Toc62220557)

[2.1 Background on Condition, Disease, or Other Primary Study Focus 10](#_Toc62220558)

[2.2 Study Rationale 11](#_Toc62220559)

[3. STUDY DESIGN 14](#_Toc62220560)

[3.1 Primary Objective 14](#_Toc62220561)

[3.2 Secondary Objectives 14](#_Toc62220562)

[3.3 Enrollment 14](#_Toc62220563)

[3.4 Blinding 15](#_Toc62220564)

[4. SELECTION AND ENROLLMENT OF PARTICIPANTS 15](#_Toc62220565)

[4.1 Inclusion Criteria 15](#_Toc62220566)

[4.2 Exclusion Criteria 15](#_Toc62220567)

[4.3 Study Enrollment Procedures 16](#_Toc62220568)

[4.4 Randomization 16](#_Toc62220569)

[5. STUDY INTERVENTIONS 17](#_Toc62220570)

[5.1 Interventions, Administration, and Duration 17](#_Toc62220571)

[5.2 Handling of Study Interventions 18](#_Toc62220572)

[5.3 Concomitant Interventions 18](#_Toc62220573)

[5.3.1 Allowed Interventions 18](#_Toc62220574)

[5.3.2 Required Interventions 18](#_Toc62220575)

[5.3.3 Prohibited Interventions 19](#_Toc62220576)

[5.4 Adherence Assessment 21](#_Toc62220577)

[6. STUDY PROCEDURES 23](#_Toc62220578)

[6.1 Schedule of Evaluations 23](#_Toc62220579)

[6.2 Description of Evaluations 26](#_Toc62220580)

[6.2.1 Procedures for Screening & Onboarding (Visit 1) 27](#_Toc62220581)

[6.2.2 Enrollment, Baseline, and Randomization 29](#_Toc62220582)

[6.2.3 Blinding 33](#_Toc62220583)

[6.2.4 Follow-up Visits 34](#_Toc62220584)

[6.2.5 Completion/Final Evaluation 39](#_Toc62220585)

[7. SAFETY ASSESSMENTS 42](#_Toc62220586)

[7.1 Specification of Safety Parameters 44](#_Toc62220587)

[7.2 Methods and Timing for Assessing, Recording, and Analyzing Safety Parameters 45](#_Toc62220588)

[7.3 Adverse Events and Serious Adverse Events 45](#_Toc62220589)

[7.4 Reporting Procedures 46](#_Toc62220590)

[7.5 Follow-up for Adverse Events 46](#_Toc62220591)

[7.6 Safety Monitoring 46](#_Toc62220592)

[8. INTERVENTION DISCONTINUATION 46](#_Toc62220593)

[9. STATISTICAL CONSIDERATIONS 47](#_Toc62220594)

[9.1 General Design Issues 47](#_Toc62220595)

[9.2 Sample Size and Randomization 49](#_Toc62220596)

[9.3 Treatment Assignment Procedures 50](#_Toc62220597)

[9.4 Interim Analyses and Stopping Rules 51](#_Toc62220598)

[9.5 Outcomes 51](#_Toc62220599)

[9.5.1 Primary Outcome 51](#_Toc62220600)

[9.5.2 Secondary Outcomes 51](#_Toc62220601)

[9.6 Data Analyses 52](#_Toc62220602)

[10. DATA COLLECTION AND QUALITY ASSURANCE 53](#_Toc62220603)

[10.1 Data Collection Forms 53](#_Toc62220604)

[10.2 Data Management 53](#_Toc62220605)

[10.3 Quality Assurance 53](#_Toc62220606)

[10.3.1 Training 53](#_Toc62220607)

[10.3.2 Quality Control Committee 55](#_Toc62220608)

[10.3.3 Metrics 56](#_Toc62220609)

[10.3.4 Protocol Deviations 56](#_Toc62220610)

[10.3.5 Monitoring 56](#_Toc62220611)

[11. PARTICIPANT RIGHTS AND CONFIDENTIALITY 57](#_Toc62220612)

[11.1 Institutional Review Board (IRB) Review 57](#_Toc62220613)

[11.2 Informed Consent Forms 57](#_Toc62220614)

[11.3 Participant Confidentiality 58](#_Toc62220615)

[11.4 Study Discontinuation 58](#_Toc62220616)

[12. COMMITTEES 58](#_Toc62220617)

[13. PUBLICATION OF RESEARCH FINDINGS 58](#_Toc62220618)

[14. REFERENCES 58](#_Toc62220619)

[15. SUPPLEMENTS/APPENDICES 61](#_Toc62220620)

# STUDY TEAM ROSTER

**Leadership**

Argye Hillis-Trupe Julius Fridriksson

Johns Hopkins University School of Medicine University of South Carolina

600 N. Wolfe Street 915 Greene Street

Phipps 446 Discovery I, 202A

Baltimore, MD 21287 Columbia, SC 29208

Email: argye@jhmi.edu Email: jfridrik@sc.edu

Phone: (410) 812-6716 Phone: (803) 777-5931

Fax: (410) 614-9807

**Participating Study Sites & Contacts**

Johns Hopkins University

School of Medicine

Melissa D. Stockbridge

600 N. Wolfe Street, Phipps 446

Baltimore, MD 21287

Email: md.stockbridge@jhmi.edu

Phone: (301) 906-2401

University of South Carolina

Leigh Ann Spell

Email: spelll@mailbox.sc.edu

915 Greene Street

Columbia, SC 29208

Phone: (803) 777-2693

Souvik Sen

8 Medical Park, Suite 420

Columbia, SC 29203

Email: Souvik.Sen@uscmed.sc.edu

Phone: (803) 545-6073

Fax: (803) 545-6051

Medical University of South Carolina

Leo Bonilha

Department of Neurology, College of Medicine

Medical University of South Carolina

68 President St, Charleston, SC 29425

Email: bonilha@musc.edu

Phone: (843) 792-5044

# PRÉCIS

**Study Title**

Escitalopram and Language Intervention for Subacute Aphasia (ELISA)

**Objectives**

Primary Objective: Conduct a Phase 2 multisite, randomized, double blind, placebo-controlled trial of escitalopram for augmenting language intervention in subacute stroke.

Primary Outcome: The change in number of correctly named items on the short form of the Philadelphia Naming Test (PNT; Walker & Schwartz, 2012) from pre-treatment to 1 week post-CoDeNT.

Secondary Objectives will test hypotheses related to the relationship between language improvement and (1.b.) antidepressant effects, (1.c.) lesion location, (2.a.) functional connectivity in the left hemisphere language network, (2.b.) connectivity in the frontolimbic circuits, and (2.c.) met alleles of BDNF.

**Design and Outcomes**

We will analyze language data from 3 sites (Johns Hopkins Medicine-JHM; University of South Carolina School of Medicine-USC; and Medical University of South Carolina-MUSC, three sites in the P50-funded Center for the Study of Aphasia Recovery, C-STAR).

Recruitment: 88 participants

Inclusion criteria:

1. Participants must have sustained an acute ischemic left hemisphere stroke.
2. Participants must be fluent speakers of English by self-report.
3. Informed consent must be obtained from the participant or legally authorized representative.
4. Participants must be age 18 or older.
5. Participants must be pre-morbidly right handed by self-report.
6. Participants must be within 5 days of onset of stroke at the time of consent.
7. Participants must have an aphasia diagnosis as confirmed by the Western Aphasia Battery-Revised (Aphasia Quotient, AQ < 93.8).

Exclusion criteria:

1. Previous neurological disease affecting the brain including previous symptomatic stroke
2. Diagnosis of schizophrenia, autism, or other psychiatric or neurological condition that affects naming/language
3. A history of additional risk factors for torsades de pointes (TdP; e.g., heart failure, hypokalemia, family history of Long QT Syndrome)
4. Current severe depression, defined as a score of > 15 on the Patient Health Questionnaire (PHQ-9) or endorsing suicidality (PHQ-9 question 9)
5. Uncorrected visual loss or hearing loss by self-report
6. Use of any medication approved by the FDA for treatment of depression at the time of stroke onset
7. Concomitant use of any monoamine oxidase inhibitors (MAOIs), pimozide and other drugs that prolong the QT/QTc interval, triptans (and other 5-Hydroxytryptamine Receptor Agonists), or other contraindications to escitalopram that may be identified. See 5.3.3. Prohibited interventions for full details.
8. A QTc greater than 450 milliseconds on electrocardiogram.
9. Evidence of hyponatremia (Na < 130) at baseline.
10. Pregnancy at the time of stroke or planning to become pregnant during the study term.

Visit summary

1. Consent and screening during acute hospitalization (0-5 days after stroke). Begin speech and language diagnostic testing.
2. Baseline assessment of naming ability (Philadelphia Naming Test short form, PNT), connected speech, and morphosyntactic production during or immediately following acute hospitalization (0-7 days after stroke).

As appropriate, participants receive functional neuroimaging, including either functional near-infrared spectroscopy (fNRIS) or structural and resting state functional magnetic resonance imaging (MRI).

At the end of Visit 2, eligible participants are randomized to receive either escitalopram or a placebo, to be taken daily for three months.

**Randomization occurs no more than 7 days post-stroke.**

1. Participants receive echocardiograms (ECG) and blood tests before, during (at Cmax), and immediately following the drug therapy period.
2. Starting 60-67 days post-randomization, participants will receive 15 45-minute sessions (Visits 4-18) of computer-delivered naming treatment (CoDeNT) over the course of 3-4 weeks.
3. Participants are evaluated at 1 week, 5 weeks, and 20 weeks post-CoDeNT on naming ability, connected speech, and morphosyntactic production. As appropriate, participants receive functional neuroimaging week 1 after the end of CoDeNT.

# 1. STUDY OBJECTIVES

## 1.1 Primary Objective

To conduct a Phase 2 multisite, randomized, double blind, placebo-controlled trial of escitalopram for augmenting language intervention in subacute stroke. This objective speaks to twin clinical goals:

1. To determine the feasibility and usefulness of moving on to a Phase III trial of escitalopram in subacute stroke, and
2. To compare the effect size of escitalopram (vs. placebo) on naming improvement to that of tDCS vs. sham (with and without SSRI) in subacute stroke.

Primary Outcome: The change in number of correctly named items on the short form of the Philadelphia Naming Test (PNT; Walker & Schwartz, 2012) from pre-treatment to 1 week post-CoDeNT.

## 1.2 Secondary Objectives

Test the following hypotheses:

Hypothesis 1.b. The effect of escitalopram on language improvement is independent of its effect on depression.

Hypothesis 1.c. The effect of escitalopram relative to placebo is significantly greater in individuals with infarcts involving left superior temporal gyrus (STG) and/or arcuate fasciculus (AF), compared to individuals without damage to left STG or AF.

Hypothesis 2.a. Greater improvement in language is associated with increased functional connectivity within the left hemisphere language network as measured by rsfMRI or fNIRS in participants who receive escitalopram, but not in those who receive placebo, independently of improvement in depression.

Hypothesis 2.b. Greater improvement in depression is associated with changes in connectivity in frontolimbic circuits in participants who received escitalopram, but not in those who receive placebo, independently of improvement in language. The goal of this exploratory aim is to determine whether or not we will be able to separate the effects of escitalopram on language and depression, using changes in network connectivity.

Hypothesis 2.c. Individuals with met alleles of BDNF show reduced response to treatment and reduced changes in connectivity in the language network, compared to those with the normal val/val alleles.

# 2. BACKGROUND AND RATIONALE

## 2.1 Background on Condition, Disease, or Other Primary Study Focus

Over two million people in the US who have survived stroke continue to struggle with everyday communication because of residual aphasia (www.aphasia.org). Language therapy is often effective in aiding language recovery after stroke. However, many hours of language intervention are required to demonstrate an effect (Bhogal et al., 2003), and most insurance providers do not cover an adequate number of treatment sessions. Therefore, there is a pressing need to improve the effectiveness and efficiency of language rehabilitation for post-stroke aphasia.

In this project, we will investigate the effects of a selective serotonin reuptake inhibitor (SSRI), escitalopram, on augmenting language therapy effectiveness, as measured when naming untrained pictures and describing pictures, in individuals with aphasia in the acute and subacute post stroke period (i.e., within three months post stroke). Naming difficulties are a persistent and common symptom in aphasia after left-hemisphere (LH) stroke. Behavioral therapy (speech and language therapy; SALT) is the mainstay treatment for post stroke aphasia (Brady et al., 2012; Kurland et al., 2012). Therapy is beneficial for language recovery in stroke, especially in the first three months post stroke (Hillis, 2010; Lazar et al., 2010). Escitalopram is a promising adjunct to traditional SALT. We recently found that survivors of left hemisphere stroke who took an SSRI daily for at least three months post-stroke showed better language recovery, compared to those who did not. (Hillis, Beh, Sebastian, et al., 2018). We first carried out a longitudinal study in which we observed that daily use of SSRIs in the first 3 months after stroke was associated with better naming recovery, independently of lesion volume, time since stroke, and depression. A large effect size for SSRI use on naming (*d* = 1.34) was observed. In the confirmatory cross-sectional study, those who took SSRIs continuously for 3 months post-stroke attained a higher accuracy on object naming than non-users (mean 85.7% vs 45.5% correct, *p* = 0.017), again with a large effect size (*d* = 1.16). (Hillis, Beh, Sebastian, et al., 2018)

However, there has been no previous RCT to evaluate the effect of daily SSRI in the first three months after stroke on improvement of language in people undergoing aphasia treatment. It is plausible that SSRIs, which elevate synaptic serotonin, might enhance recovery by augmenting synaptic plasticity. Although several groups have evaluated the effects of pharmacotherapy (plus SALT) on improving language after post-stroke aphasia, most previous studies have been carried out in chronic stroke, usually more than 1 year after stroke. The few studies of pharmacotherapy for aphasia that have shown substantial positive results have studied medical intervention in the subacute period after stroke. (Enderby et al., 1994; Huber et al., 1997) Because neuroplasticity is greatest early after stroke, there is reason to believe therapeutic SSRI might be most effective in the acute-subacute period. We will evaluate the proposed mechanism of SSRI on neural mechanisms underlying the effects of an SSRI on augmenting language recovery using longitudinal resting state fMRI (rsfMRI) and genetic testing.

To supplement the fMRI data, we will also collect task-based and task-free (“resting”) functional Near-Infrared Spectroscopy (fNIRS) data. The fNIRS protocol is expected to take up to 60 minutes. fNIRS is a safe, non-invasive, and flexible modality for brain imaging. It capitalizes on the fact that near-infrared light can propagate several centimeters through tissue because of low optical absorption by hemoglobin and water at specific wavelengths. During an fNIRS experiment, an array of light sources and detectors affixed to a cap is placed on the scalp, and the measures from these different channels allow the reconstruction of an image of the hemodynamic response. fNIRS has emerged as a complementary technology to other brain imaging and monitoring modalities (e.g., EEG, fMRI). Previous research (Huppert et al., 2006; Strangman et al., 2002) in healthy populations has shown that the hemodynamic response captured in fNIRS is similar to that measured in fMRI. Collecting either fMRI or fNIRS data at each time point in the present study will allow us to recruit participants for whom MRI is contraindicated and will provide greater flexibility to complete assessments outside of the hospital. Similar to task-free fMRI, “resting” fNIRS will allow us to determine intrinsic functional connectivity between regions of the brain at each stage of stroke recovery. In the event of a pandemic or other event that prevents participants from having MRI, we will be able to use fNIRS to test hypotheses about changes in connectivity associated with treatment, as the fNIRS equipment is not only highly portable, but can also be disinfected relatively easily after each use.

We propose to conduct a Phase 2 multisite, randomized, double blind, placebo-controlled trial of escitalopram for augmenting language intervention in subacute stroke. We hypothesize that daily escitalopram for 90 days after stroke results in greater improvement (compared to placebo) in naming untrained pictures, as well as greater increase in content of picture description and greater improvement in morphosyntactic production, when combined with SALT. A second aim is to evaluate the mechanisms of language recovery in individuals who receive active medical treatment and those who receive placebo, using functional imaging (rsfMRI & fNIRS) and genetic testing. We hypothesize that greater improvement in language is associated with increased connectivity within the left hemisphere language network on functional imaging measures in participants who receive escitalopram than in those who receive placebo, independently of improvement in depression. We also hypothesize that the effects are greatest in individuals with val/val allele of BDNF (consistent with previous studies showing a greater response to treatment and greater neuroplasticity in people with the val/val allele than those with one or more met alleles (Fridriksson et al., 2018; Wang et al., 2014).

## 2.2 Study Rationale

A large, placebo-controlled, randomized clinical trial (RCT) demonstrated that a selective serotonin reuptake inhibitor (SSRI), fluoxetine, results in better motor recovery compared to placebo, if taken daily in the first three months post-stroke. (Chollet et al., 2011) The effect of fluoxetine on motor recovery was independent of effects on depression. Another RCT demonstrated improved cognitive recovery associated with SSRI use after stroke. (Jorge et al., 2010; see also Pan et al., 2018 for similar results in an open-label trial). However, two recent RCTs showed no significant effect of SSRI use on functional outcome after stroke, measured with the Modified Rankin Scale (mRS) (Kraglund, et al., 2018; Dennis et al., 2018). The mRS is a 5 point scale that is heavily weighted toward motor recovery (e.g. gait). Nevertheless, a recent meta-analysis of eight trials showed significant effects of SSRI use on functional outcome (measured with the NIHSS and the Barthel Index) (Gu et al., 2018; see also Mead, et al., 2012). Therefore, there remains equipoise regarding the effect of SSRIs on motor recovery. In contrast, the mRS is insensitive to changes in language, other than complete recovery from aphasia (mRS=0) versus some degree of aphasia at outcome (mRS 1-4, depending on other functional deficits). We recently found that survivors of left hemisphere stroke who took an SSRI daily for at least three months post-stroke showed better language recovery compared to those who did not. (Hillis, Beh, Sebastian et al., 2018) The effect on language improvement was independent of the effect on depression, and it was greatest in those with lesions including left STG and/or AF. However, there has been no previous RCT to evaluate the effect of daily SSRI in the first three months after stroke on improvement of language in people undergoing aphasia treatment. This proposed study will allow us to compare the effect size of a daily SSRI use to the effect size of tDCS (in the ongoing SLISSE trial), combined with the identical SALT 1 week post-CoDeNT.

Recent studies in the Center for the Study of Language Recovery (C-STAR), supported by the current funding cycle of this P50, have demonstrated that the brain-derived neurotrophic factor (BDNF) gene modulates the effects of tDCS. (Fridricksson et al., 2018) Data from an RCT of anodal tDCS confirmed that outcome is influenced by an interaction between anodal-tDCS and a single-nucleotide polymorphism of the BDNF gene, rs6265. That is, participants with the normal val/val genotype who received anodal-tDCS showed greater response to aphasia treatment than val/val participants who received sham tDCS and showed greater response to aphasia treatment than the met allele carriers, regardless of tDCS condition. In other studies, the met allele has been associated with abnormal functional connectivity in both healthy controls (Wang et al., 2014) and people with depression (Doron et al., 2014. Both we (Sebastian et al., 2017) and others (Marangalo et al., 2016) have shown that language improvement with treatment after stroke is associated with changes in functional connectivity. The presence of a BDNF met allele may hinder the changes in functional connectivity needed for language recovery.

In our ongoing trial on the effects of tDCS vs sham plus computer-delivered naming therapy (CoDeNT) on aphasia recovery in subacute stroke, we evaluated the effects of SSRI use (with or without tDCS as we are still masked to treatment condition) on language recovery in the first 22 completers of the study. SSRI use depended on physician’s discretion. We found no significant differences between SSRI users and nonusers in age, education, or any test at baseline. Together, SSRI use, depression score (on the PHQ-9), age, and education predicted highest quartile of change in Content Units (CU) in picture description; pseudo r^2^ = 0.62 (p=0.016). SSRI users were more likely to achieve the highest quartile in CU (OR: 230; 95%CI: 1.4-39170), compared to nonusers. Although there were no significant differences between SSRI users and nonusers in change on any of the tests (likely due to low power), SSRI users showed higher mean improvements on all tests compared to nonusers; CU: 6.5 vs 2.6; PNT: 39.2 vs. 35.9; HANA: 7.4 vs 1.8; Cinderella Story: 12.1 vs 10.9. (Hillis, 2019)

These data provide pilot evidence that it is possible to recruit, test, and treat participants within 3 months of stroke onset. They also provide preliminary evidence that SSRIs in the first 3 months may augment language therapy for aphasia. This result complements our previously reported findings from a study of 45 people with chronic post-stroke aphasia, administered the Western Aphasia Battery (WAB). Results showed that Highest Quartile on the WAB Summary Score (omitting the subjective rating scales) was predicted by a model that included education, age, infarct volume, and antidepressant use (chi square for goodness of fit=207; p<0.0001; Cox and Snell r^2^=0.47). Antidepressant use was significantly associated with highest quartile WAB summary score independently of the other variables (Wald=5.2; p=0.022). Compared to those who had never taken antidepressants, those taking antidepressants had higher repetition scores (mean 9.4 vs.7.6; p=0.039), even though they had larger strokes (mean 225 vs 82 cc; p=0.008); the 2 groups were not different in age, education, or time post-stroke. (Hillis & Tippett, 2014).

# 3. STUDY DESIGN

## 3.1 Primary Objective

To conduct a Phase 2 multisite, randomized, double blind, placebo-controlled trial of escitalopram for augmenting language intervention in subacute stroke. This objective speaks to twin clinical goals:

1. To determine the feasibility and usefulness of moving on to a Phase III trial of escitalopram in subacute stroke, and
2. To compare the effect size of escitalopram (vs. placebo) on naming improvement to that of tDCS vs. sham (with and without SSRI) in subacute stroke.

Primary Outcome: The change in number of correctly named items on the short form of the Philadelphia Naming Test (PNT; Walker & Schwartz, 2012) from pre-treatment to 1 week post-CoDeNT.

## 3.2 Secondary Objectives

Test the following hypotheses:

Hypothesis 1.b. The effect of escitalopram on language improvement is independent of its effect on depression.

Hypothesis 1.c. The effect of escitalopram relative to placebo is significantly greater in individuals with infarcts involving left superior temporal gyrus (STG) and/or arcuate fasciculus (AF), compared to individuals without damage to left STG or AF.

Hypothesis 2.a. Greater improvement in language is associated with increased functional connectivity within the left hemisphere language network as measured by rsfMRI or fNIRS in participants who receive escitalopram, but not in those who receive placebo, independently of improvement in depression.

Hypothesis 2.b. Greater improvement in depression is associated with changes in connectivity in frontolimbic circuits in participants who received escitalopram, but not in those who receive placebo, independently of improvement in language. The goal of this exploratory aim is to determine whether or not we will be able to separate the effects of escitalopram on language and depression, using changes in network connectivity.

Hypothesis 2.c. Individuals with met alleles of BDNF show reduced response to treatment and reduced changes in connectivity in the language network, compared to those with the normal val/val alleles.

## 3.3 Enrollment

Recruitment is expected to occur in acute post-stroke inpatient units (N=88), with the expectation that participants continue study activities after discharge for a total of 7-8 months. Participation in this study will not disrupt any current care or therapy.

## 3.4 Blinding

The study is to be conducted in a double-blind manner. The subjects, the site investigators, and the clinical staff involved in this study will not know the treatment assignment. The statistical team at the Statistical and Data Management Center will be unblinded. The study statistician will provide a sealed envelope with the treatment group identifiers to the DSMB. This envelope would only be opened by the DSMB if they require unblinding or at the end of the study.

# 4. SELECTION AND ENROLLMENT OF PARTICIPANTS

## 4.1 Inclusion Criteria

Patients must satisfy the following inclusion criteria to be considered eligible for entry into this study:

1. Participants must have sustained an acute ischemic left hemisphere stroke.
2. Participants must be fluent speakers of English by self-report.
3. Participants must be capable of giving informed consent or indicating a legally authorized representative to provide informed consent.
4. Participants must be age 18 or older.
5. Participants must be pre-morbidly right handed by self-report.
6. Participants must be within 5 days of onset of stroke.
7. Participants must have an aphasia diagnosis as confirmed by the Western Aphasia Battery-Revised (AQ < 93.8).

## 4.2 Exclusion Criteria

Patients with any of the following characteristics will not be eligible for entry into this study:

1. Previous neurological disease affecting the brain including previous symptomatic stroke
2. Diagnosis of schizophrenia, autism, or other psychiatric or neurological condition that affects naming/language
3. A history of additional risk factors for torsades de pointes (TdP; e.g., heart failure, hypokalemia, family history of Long QT Syndrome)
4. Current severe depression, defined as a score of > 15 on the Patient Health Questionnaire or endorsing suicidality (PHQ-9 question 9)
5. Uncorrected visual loss or hearing loss by self-report
6. Use of any medication approved by the Food and Drug Administration (FDA) for treatment of depression at the time of stroke onset
7. Concomitant use of any monoamine oxidase inhibitors (MAOIs), pimozide and other drugs that prolong the QT/QTc interval, triptans (and other 5-Hydroxytryptamine Receptor Agonists), or other contraindications to escitalopram that may be identified. See 5.3.3. Prohibited interventions for full details.
8. A QTc greater than 450 milliseconds on electrocardiogram or evidence of hyponatremia (Na < 130) at baseline.
9. Pregnancy at the time of stroke or planning to become pregnant during the study term.

## 4.3 Study Enrollment Procedures

Recruitment will take place in inpatient units by trained study team members. Stroke patients will be recruited from inpatient stroke facilities. All three sites have comprehensive stroke units; MUSC is similar in size to JHM, and USC is somewhat smaller. We expect to randomize approximately 7 participants per year at each site. Study team members are told about the patients by the inpatient attending physician or resident caring for the patient (see HIPAA waiver, and why the study could not practicably be carried out without this waiver). Patients are approached by the study team member and assessed for comprehension with questions from an aphasia battery. If they have impaired comprehension, the Legally Authorized Representative (LAR) is contacted about the study for consent to participate.

All study team members have experience working with individuals with impaired language and cognition. We will make every effort to make the study understandable to the patient, by speaking in simple, short sentences with low-level vocabulary. We will assess comprehension by asking them questions about their understanding of the study. For patients with impaired comprehension, we will obtain assent from the patient and consent from the Legally Authorized Representative (LAR). We will explain the study and obtain consent in a room with a closed door.

All participants (or their Legally Authorized Representative) are asked to sign a consent form and no record of their history is available to anybody except from the study team members.

Documentation of reasons for ineligibility and non-participation of eligible candidates will be reported in the trial database.

We will go through the consent process again with participants with any comprehension problems, or with their Legally Authorized Representative (LAR) when they return for follow-up to ensure continued understanding of their participation.

## 4.4 Randomization

The randomization will take place centrally via the Trial Website. Subjects will be randomized 1:1 (escitalopram or placebo), controlling for severity (classified using the Western Aphasia Battery revised: WAB-R). The computer program developed at the DCU makes the treatment assignment based on the current status of treatment group distribution within each stratum as well as overall balance of treatment assignment. The detailed randomization scheme and source codes will be provided in the Randomization Plan document.

A “Real-Time” randomization procedure is implemented via the Trial Website on the WebDCU^TM^ System where the clinical center staff enters the basic baseline (e.g. AQ severity) and eligibility information of a subject prior to randomization. If the subject’s eligibility status is confirmed, the computer program on the WebDCU^TM^ server will evaluate the treatment arm distribution and generate a blinded randomization code on the randomization scheme. The speech language pathologist (SLP) or research team member randomizing the patient will not know the treatment assignment. The pharmacy at the participating center will be notified of the randomization code, refer to the randomization code list provided by NDMC, and dispense the active drug or placebo from their inventory.

# 5. STUDY INTERVENTIONS

## 5.1 Interventions, Administration, and Duration

Escitalopram

Escitalopram is a well-tolerated SSRI that has been shown to be effective in treatment of depression and anxiety at a dosage of 10 mg per day. The FLAME trial found that fluoxetine 20 mg per day for 3 months after stroke onset was associated with greater motor recovery compared to placebo. The equivalent dose of escitalopram is 9 mg per day. (Hayasaka et al., 2015). Escitalopram is commercially available at a dose of 10 mg, and this is the recommended therapeutic dosage for individuals 65 and older; so we will use 10 mg as the study dose. This dose was also used in the RCT of escitalopram to improve cognitive function post-stroke (Jorge et al., 2010).

Escitalopram has been associated with spontaneous reports of adverse events occurring upon discontinuation, particularly when abrupt, including the following: dysphoric mood, irritability, agitation, dizziness, sensory disturbances (e.g., paresthesias such as electric shock sensations), anxiety, confusion, headache, lethargy, emotional lability, insomnia, and hypomania. Therefore, participants will be up-titrated onto the medication by taking 5 mg escitalopram (or placebo) for the first week of treatment and tapered off the medication by taking 5 mg escitalopram (or placebo) for 2 weeks after the 90 days of treatment.

Antidepressants, such as escitalopram, increased the risk compared to placebo of suicidal thinking and behavior (suicidality) in children, adolescents, and young adults in short-term studies of major depressive disorder (MDD) and other psychiatric disorders. However, there was a reduction in risk with antidepressants compared to placebo in adults aged 65 and older. Participants with antidepressant use at the time of stroke onset or moderately severe depression (PHQ-9 score >15) at enrollment are excluded from the present study to minimize the possibility that individuals with MDD will be enrolled in the study.

The most common side effects are drowsiness, nausea, insomnia, dry mouth, constipation, which are also common with placebo. A rare, but more serious side effect is prolongation of the QT interval (QTc) on electrocardiogram, which can lead to a hazardous arrhythmia. In healthy individuals, the mean QTc is approximately 400 ms (up to 450 ms for men and 460 ms for women). A QTc longer than 500 ms or an increase in QTc greater than 60 ms is considered a major risk factor for dangerous arrhythmia. (Lam, 2013).

Participants who develop a QTc > 450 ms or an increase > 60 ms over baseline, or other serious arrhythmia (identified by the consulting cardiologist at JHMI, Dr. David Spragg), or those who develop moderate-to-severe depression (PHQ-9 >15) will be withdrawn from the study. We will exclude people with contraindications to escitalopram, including medications with serious interactions.

An IND is not required as this Phase II trial is not designed to change indications for the FDA approved medication, escitalopram.

Computer Delivered Naming Treatment (CoDeNT)

Participants in both groups will receive the same 15 45-minute sessions of a computer-delivered naming treatment (CoDeNT). Speech and language treatment is the current clinical standard of care for post-stroke aphasia. Sessions will be completed online facilitated by video conferencing software (Zoom). The 15 sessions of CoDeNT will be provided in addition to any ongoing speech and language treatment. We will record the frequency, session duration, and number of sessions of language therapy received by each participant as co-variants but will not attempt to control language treatment other than the 15 sessions of CoDeNT.

## 5.2 Handling of Study Interventions

We have identified a supplier (at Temple University) of a placebo that is indistinguishable from a generic escitalopram tablet; the placebo will be supplied in bulk to the research pharmacy at each site. Each site’s investigational pharmacy will order the active drug in bulk from their own catalogue (Teva or Solco brand). The medication or placebo will be dispensed by each institution’s research pharmacy from a pre-packaged and pre-registered set of allotments established for each year. The medication or placebo will be stored, prepared, dispensed, and destroyed per the standard operating procedures of each institution’s research pharmacy.

Accountability: medication adherence will be measured by counting pills at each visit (during CoDeNT) and contacting the participant/caregiver daily during hospitalization and weekly during any period the participant is not hospitalized prior to beginning treatment with CoDeNT.

## 5.3 Concomitant Interventions

### 5.3.1 Allowed Interventions

Participants will be expected to continue with any existing medications prescribed by a physician and any supplements available over-the-counter, as managed by their primary physician.

### 5.3.2 Required Interventions

No additional interventions are required.

### Prohibited Interventions

At the time of stroke onset, prohibited interventions are pimozide and other drugs that prolong the QT/QTc interval, triptans (and other 5-Hydroxytryptamine Receptor Agonists), any medication approved by the FDA for treatment of depression, or other contraindications to escitalopram that may be identified. All subjects will be given a list of drugs to avoid during the course of the study. If a participant starts a medication that is contraindicated, they will stop study medication but continue to be followed through the end of the study.

Approved for the treatment of depression

- Selective Serotonin Reuptake Inhibitors (SSRIs)
  - Escitalopram (Lexapro)
  - Citalopram (Celexa)
  - Paroxetine (Paxil, Paxil CR, Pexeva)
  - Fluoxetine (Prozac, Symbyax)
  - Vortioxetine (Trintellix)
  - Vilazodone (Viibryd)
  - Sertaline (Zoloft)
- Serotonin and Norepinephrine Reuptake Inhibitors (SNRIs)
  - Duloxetine (Cymbalta)
  - Venlafaxine (Effexor)
  - Levomilnacipran (Fetzima)
  - Desvenlafaxine (Pristiq, Khedezla)
- Monoamine Oxidase Inhibitors (MAOIs)
  - Selegiline (Emsam, skin patch)
  - Isocarboxzaid (Marplan)
  - Phenelzine (Nardil)
  - Tranylcypromine (Parnate)
- Tricyclic/Tetracyclic Antidepressants
  - Amoxapine (Ascendin)
  - Amitriptyline (Elavil)
  - Desipramine (Norpramin)
  - Nortriptyline (Pamelor)
  - Doxepin (Sinequan)
  - Trimipramine (Surmontil)
  - Imipramine (Tofranil)
  - Portriptyline (Vivactil)
  - Maprotiline (Ludiomil)
- Atypical Antidepressants
  - Trazodone (Desyrel)
  - Nefazodone (Serzone)
  - Mirtazapine (Remeron)
  - Bupropion (Wellbutrin)
- NMDA Antagonists
  - Esketamine (Spravato, nasal spray)
- Neuroactive Steroid GABA-A Receptor Positive Modulator
  - Brexanolone (Zulresso, IV)

More than twice weekly use of triptans approved for the treatment of moderate-severe migraine

- Almotriptan (Axert)
- Eletriptan (Relpax)
- Frobatriptan (Frova)
- Naratriptan (Amerge)
- Rizatriptan (Maxalt)
- Sumatriptan (Imitrex)
- Zolmitriptan (Zomig)

Daily use of the following medications approved for the treatment of bipolar disorder

- Atypical Antipsychotics
  - Aripiprazole (Abilify)
  - Asenapine (Saphris)
  - Cariprazine (Vraylar)
  - Lurasidone (Latuda)
  - Olanzapine (Zyprexa, Symbyax)
  - Quetiapine (Seroquel)
  - Risperidone (Risperdal)
  - Ziprasidone (Geodon)
- Salts
- Lithium

Additional drugs known to prolong the QT/QTc interval

- Antianginal
  - Bepridil (Vascor)
- Antiarrythmics
  - Amiodarone (Cordarone, Pacerone, Nexterone)
  - Disopyramide (Norpace)
  - Dofetilide (Tikosyn)
  - Dronedarone (Multaq)
  - Flecanide (Tambocor, Almarytm, Apocard, Ecrinal, Flécaine)
  - Ibutilide (Covert)
  - Procainamide (Pronestyl, Procan)
  - Quinidine (Quinaglute, Duraquin, Quinact, Quinidex, Cin-Quin, Quinora)
  - Sotalol (Betapace, Sotalex, Sotacor)
- Antibiotics
  - Azithromycin (“Z-pack,” Zithromax, Zmax)
  - Ciprofloxacin (Cipro, Neofloxin)
  - Clarithromycin (Biaxin, Prevpac)
  - Levofloxacin (Levaquin, Tavanic)
  - Moxifloxacin (Avelox, Avalox, Avelon)
  - Erythromycin (E.E.S., Robimycin, EMycin, Erymax, Ery-Tab, Eryc Ranbaxy, Erypar, Eryped, Erythrocin Stearate Filmtab, Erythrocot, E-Base, Erythroped, Ilosone, MY-E, Pediamycin, Abboticin, Abboticin-ES, Erycin, PCE Dispertab, Stiemycine, Acnasol, Tiloryth)
- Antiemetics
  - Chlorpromazine (Thorazine, Largactil, Megaphen)
  - Ondansetron (Zofran, Anset, Ondemet, Zuplenz, Emetron, Ondavell, Emeset, Ondisolv, Setronax)
- Antifungals
  - Fluconazole (Diflucan, Trican)
  - Pentamidine (Pentam)
- Antimalarials
  - Chloroquine (Aralen)
  - Hydroxychloroquine (Plaquenil, Quineprox)
- Antipsychotics
  - Thioridazine (Mellaril, Novoridazine, Thioril)
  - Haloperidol (Haldol, Aloperidin, Bioperidolo, Brotopon, Dozic, Duraperidol, Einalon S, Eukystol, Halosten, Keselan, Linton, Peluces, Serenace, Serenase, Sigaperidol)
  - Pimozide (Orap)
- Drugs used in the treatment of certain cancers
  - Oxaliplatin (Eloxatin)
  - Arsenic trioxide (Trisenox)
  - Vandetanib (Caprelsa)
- Drugs used in the treatment of dementia
  - Donepezil (Aricept)
- Drugs used in the treatment of intermittent claudication
  - Cilostazol (Pletal)
- Drugs used in the treatment of thrombocythemia
  - Anagrelide (Agrylin, Xagrid)
- Schedule II substances
  - Cocaine
  - Methadone

If a participant must begin one of the prohibited interventions as part of their routine healthcare outside of the study, this will cause the participant to stop study medication but continue to be followed through the end of the study.

## Adherence Assessment

To ensure compliance, we will count pills at each visit (during CoDeNT) and contact the participant/caregiver daily during hospitalization and weekly during any period the participant is not hospitalized prior to beginning treatment with CoDeNT. Other than monitoring and data collection required to be completed in person (i.e., ECG, blood electrolyte testing, neuroimaging, grip strength and peg board), all other visit activities will be completed remotely via online video meeting software.

Adherence will be defined as at least 85% of treatment intervention pills taken (operationalized as the difference between the number remaining and number supplied proportionate to the number supplied), AND at least 85% of CoDeNT sessions attended (as documented by the therapist). Pills missing from the participant’s ongoing supply at each count are presumed to have been taken by the participant unless the participant states otherwise. Visits are considered attended if the participant completes the treatment activities for that session, as determined by the therapist administering the session.

# 6. STUDY PROCEDURES

## 6.1 Schedule of Evaluations

| Assessment | Screening & Onboarding  Visit 1  Post-stroke day 0-5 | Baseline Evaluation & Randomization  Visit 2  Post-stroke day 0-7 | Weekly from randomization  until drug treatment end (post-randomization day 104) | Visit 3  14 days from randomization + 0-7 days | CoDeNT Naming Treatment  Visits  4-18  Begins 60 days from randomization + 0-7 days  Ends by post-randomization day 90 | Visit 8  Between 60-90 days post-randomization | Visit 13  Between 60-90 days post-randomization | Visit 18  Between 60-90 days post-randomization | Post-CoDeNT  Evaluation  (1 week)  Visit  19  Minimum: post-randomization day 93  Maximum: post-randomization day 104 | Post-CoDeNT Evaluation (5 weeks)  Visit  20  Minimum: post-randomization day 116  Maximum: post-randomization day 132 | Post-CoDeNT Evaluation  (20 weeks) Visit  21  Minimum: post-randomization day 221  Maximum: post-randomization day 237 |
| --- | --- | --- | --- | --- | --- | --- | --- | --- | --- | --- | --- |
| Informed consent | X |  |  |  |  |  |  |  |  |  |  |
| Review inclusion/exclusion criteria | X |  |  |  |  |  |  |  |  |  |  |
| Medical history | X |  |  |  |  |  |  |  |  |  |  |
| Current medications | X |  |  |  |  |  |  |  |  |  |  |
| Social history | X |  |  |  |  |  |  |  |  |  |  |
| Neurological Exam (with NIHSS) | X |  |  |  |  | X | X | X |  | X | X |
| MRI safety screening (if applicable) | X |  |  |  |  |  |  |  |  |  |  |
| Western Aphasia Battery |  | X |  |  |  |  |  |  |  |  |  |
| Boston Naming Test |  | X |  |  |  |  |  |  |  |  |  |
| Modified Rankin Scale |  | X |  |  |  |  |  |  | X | X | X |
| Stroke Aphasia Quality of Life Scale-39 |  | X |  |  |  |  |  |  | X | X | X |
| Assessment | Screening & Onboarding  Visit 1  Post-stroke day 0-5 | Baseline Evaluation & Randomization  Visit 2  Post-stroke day 0-7 | Weekly from randomization  until drug treatment end (post-randomization day 104) | Visit 3  14 days from randomization + 0-7 days | CoDeNT Naming Treatment  Visits  4-18  Begins 60 days from randomization + 0-7 days  Ends by post-randomization day 90 | Visit 8  Between 60-90 days post-randomization | Visit 13  Between 60-90 days post-randomization | Visit 18  Between 60-90 days post-randomization | Post-CoDeNT  Evaluation  (1 week)  Visit  19  Minimum: post-randomization day 93  Maximum: post-randomization day 104 | Post-CoDeNT Evaluation (5 weeks)  Visit  20  Minimum: post-randomization day 116  Maximum: post-randomization day 132 | Post-CoDeNT Evaluation  (20 weeks) Visit  21  Minimum: post-randomization day 221  Maximum: post-randomization day 237 |
| Positive and Negative Affect Schedule-SF |  | X |  |  |  |  |  |  | X | X | X |
| Multidimensional Scale of Perceived Social Support |  | X |  |  |  |  |  |  |  |  | X |
| Life Events Checklist-5 |  | X |  |  |  |  |  |  |  |  | X |
| Big Five Inventory-2 Ne items |  | X |  |  |  |  |  |  |  |  |  |
| General Anxiety Disorder-7 |  | X |  |  |  |  |  |  |  |  |  |
| Brief Resilient Coping Scale |  | X |  |  |  |  |  |  |  |  |  |
| Morphosyntactic Generation Test |  | X |  |  |  |  |  |  | X |  |  |
| Cinderella retelling |  | X |  |  |  |  |  |  | X | X | X |
| Cookie Theft picture description |  | X |  |  |  |  |  |  |  | X | X |
| Philadelphia Naming Test, short form (x2) |  | X |  |  |  |  |  |  | X | X | X |
| **Grip strength test** | **X** |  |  |  |  |  |  |  | **X** |  |  |
| **9-peg board test** | **X** |  |  |  |  |  |  |  | **X** |  |  |
| **Saliva sample for BDNF testing** | **X** |  |  |  |  |  |  |  |  |  |  |
| Assessment | Screening & Onboarding  Visit 1  Post-stroke day 0-5 | Baseline Evaluation & Randomization  Visit 2  Post-stroke day 0-7 | Weekly from randomization  until drug treatment end (post-randomization day 104) | Visit 3  14 days from randomization + 0-7 days | CoDeNT Naming Treatment  Visits  4-18  Begins 60 days from randomization + 0-7 days  Ends by post-randomization day 90 | Visit 8  Between 60-90 days post-randomization | Visit 13  Between 60-90 days post-randomization | Visit 18  Between 60-90 days post-randomization | Post-CoDeNT  Evaluation  (1 week)  Visit  19  Minimum: post-randomization day 93  Maximum: post-randomization day 104 | Post-CoDeNT Evaluation (5 weeks)  Visit  20  Minimum: post-randomization day 116  Maximum: post-randomization day 132 | Post-CoDeNT Evaluation  (20 weeks) Visit  21  Minimum: post-randomization day 221  Maximum: post-randomization day 237 |
| **Functional imaging (fNIRS or MRI)** | **X** |  |  |  |  |  |  |  | **X** |  |  |
| **Electrocardiogram** | **X** |  |  | **X** |  |  |  |  | **X** |  |  |
| **Blood test (electrolytes)** | **X** |  |  | **X** |  |  |  |  | **X** |  |  |
| Enrollment/Randomization |  | X |  |  |  |  |  |  |  |  |  |
| Patient Health Questionnaire 9  (weekly on Monday) | X | X | X |  | X |  |  |  | X |  |  |
| Count pills or review medication administration records |  |  | X | X | X |  |  |  |  |  |  |
| Blood Pressure |  |  |  |  | X |  |  |  |  |  |  |
| Heart Rate |  |  |  |  | X |  |  |  |  |  |  |
| Adverse Events |  |  | X | X | X |  |  |  | X | X | X |

**Assessments in BOLD must be completed in person at the participant’s recruitment at site (JHU, MUSC, or USC).**

6.2 Description of Evaluations
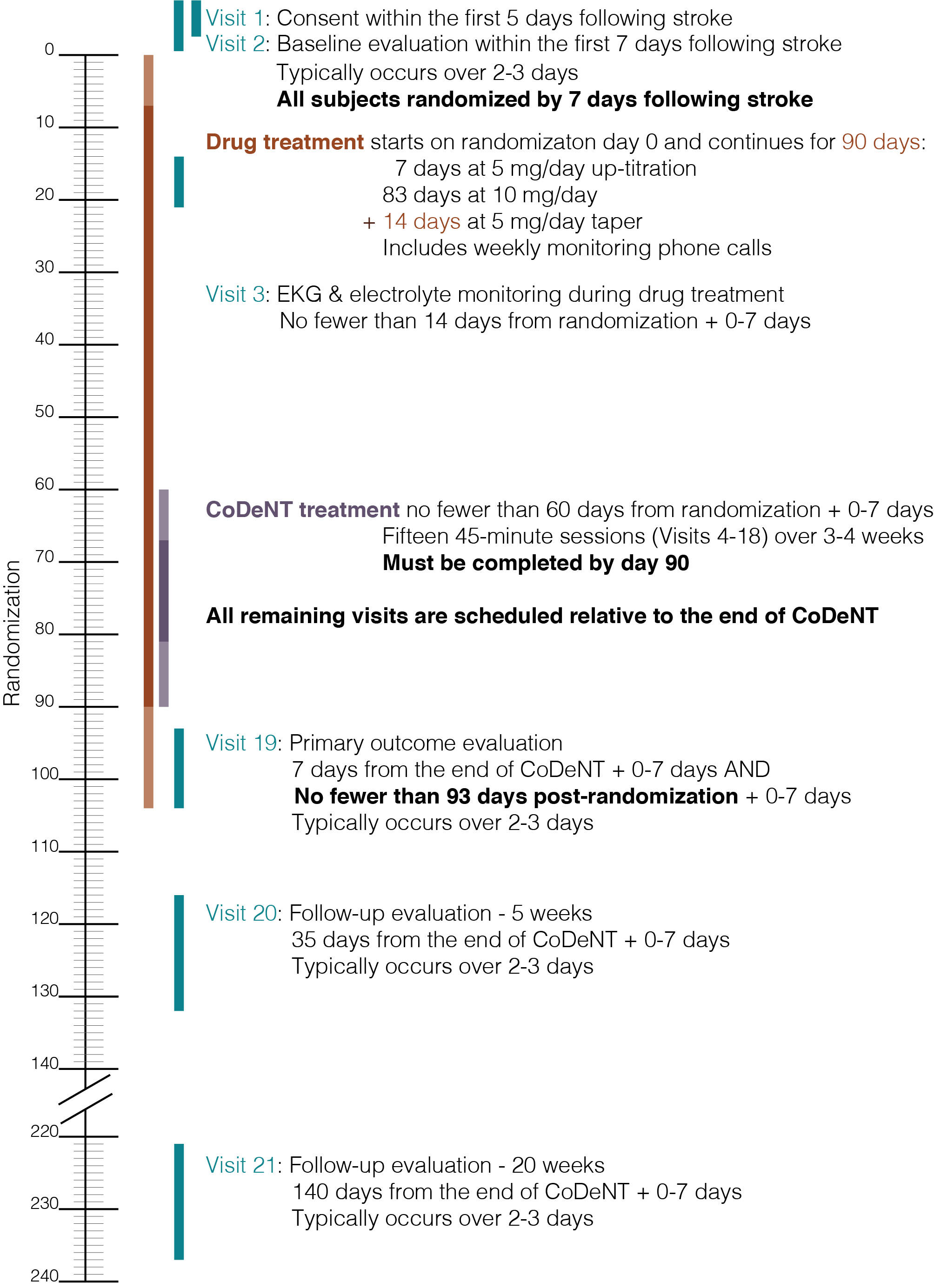


### 6.2.1 Procedures for Screening & Onboarding (Visit 1)

Visit 1 will be performed **within 5 days** of stroke onset, typically during acute hospitalization, and **requires direct contact to complete neuroimaging (if elected), genetic sampling (if elected), ECG, blood testing, and measures of motor status (grip strength and peg board).** Additional activities in Visit 1 may be completed either in person or remotely to reduce the risks associated with prolonged participant-researcher contact.

**Note: The events of Visit 1 are completed on-site at each recruitment location.**

**The following procedures will be performed:**

1. Consenting Procedure
   1. A signed and dated informed consent form will be obtained from each participant before conducting any screening procedures. Consent procedures may be completed via video conferencing as necessary to reduce participant-researcher contact. Participants and/or their legally authorized representative (LAR) will be provided with the consent forms to review. Research staff will then participate in a video call with the consenting party to review the form and answer questions. Participants may elect not to participate in the genetic testing, MRI, or fNIRS procedures of the study and still complete the drug trial. Participants will be then be assigned a temporary identification number for the purposes of initial screening.
   2. All research staff authorized to obtain informed consent will have completed the Miami CITI course in the Responsible Conduct of Research and Protection of Human Subjects prior to their involvement with the study. Furthermore, they will be oriented to the study and trained by the study PI and study co-investigators who have all had extensive training and experience in the ethical and practical aspects of informed consent procedures.
2. Review inclusion/exclusion criteria
3. Obtain medical history, including medications and social history. All subjects will be given a list of drugs to avoid during the course of the study. See 5.3.3. Prohibited interventions for full details. If a participant starts a medication that is contraindicated (as specified in the exclusion criteria), they will stop study medication but continue to be followed through the end of the study.
4. Conduct neurological examination either in person or remotely via video call with a neurologist, including the NIH Stroke Scale (NIHSS; Lyden, 1999).
   1. Clinicians will be certified in administration of the NIHSS by taking a web-based course offered by the American Heart Association.
   2. The NIHSS will provide a measure of overall stroke severity. The SLP will also record the description of the “Cookie Theft” picture on the NIHSS, which will also be analyzed for Content Units (CU) and Syllable/CU (Yorkston & Beukelman, 1980). This test will be repeated at Week 5 and Week 20 after the end of CoDeNT.
5. Administer the MRI safety screening (if applicable)
6. Administer Patient Health Questionnaire 9 (depression screening questionnaire)

**A portion of this visit must be completed in person, which includes the following activities:**

1. Obtain saliva (cheek swab) for BDNF testing.
   1. Kits will be received from and returned to JHU for processing
2. Conduct functional imaging baseline evaluation. This will include either MRI or fNIRS.
   1. Structural and rsfMRI will be acquired in the hospital.
      1. Run the participant on a 10-minute resting state functional connectivity MRI exam during visits two and immediately after treatment periods 1 and 2:
      2. Instruct the participant to lie still in the scanner, eyes open
      3. Run the participant on high-resolution anatomical MRI scans during each of the same visits
      4. Instruct the participant to lie still during these scans
   2. fNIRS will be conducted in a clinical space (i.e., hospital or outpatient clinic).
3. Grip strength and 9-peg board tests
4. Obtain electrocardiogram or collect results from hospitalization record and email to David Spragg (JHU) for central interpretation.
5. Check electrolytes (blood test) or collect results from hospitalization record

**Screening Checklist**

- Within 5 days of acute left hemisphere ischemic stroke evidenced by imaging (CT, MRI) at the time of consent
- Fluent speaker of English by self-report
- 18 or older
- Pre-morbidly right handed by self-report
- Diagnosis of aphasia on Western Aphasia Battery-R (Aphasia Quotient < 93.8, completed during Visit 2)
- No history of neurological disease, including previous symptomatic stroke
- No prior diagnosis of schizophrenia, autism, or other psychiatric or neurological condition that affects naming/language
- No history of additional risk factors for TdP (e.g., heart failure, hypokalemia, family history of Long QT Syndrome)
- Not currently in a state of severe depression (PHQ > 15) or endorsing suicidality (PHQ-9 question 9)
- No uncorrected visual or hearing loss by self-report
- Not taking any medication approved by the FDA for the treatment of depression at the time of stroke onset
- Not taking a monoamine oxidase inhibitors (MAOIs), pimozide and other drugs that prolong the QT/QTc interval, triptans (and other 5-Hydroxytryptamine Receptor Agonists), or other contraindications to escitalopram that may be identified. See 5.3.3. Prohibited interventions for full details.
- Not exhibiting QTc greater than 450 milliseconds on electrocardiogram or evidence of hyponatremia Na < 130
- Not currently pregnant or planning to become pregnant during the study term

### 6.2.2 Enrollment, Baseline, and Randomization

#### **Enrollment**

Patients who meet all criteria on the Screening Checklist will be provided the opportunity to provide informed consent. Patients or their legally authorized representative (LAR) will be invited to sign a single informed consent form that includes both screening and study procedures. If the participant passes the screening portion, diagnostic testing will be conducted during the same visit or during successive remote sessions within the next week.

#### **Procedures for Baseline Testing (Visit 2)**

Visit 2 occurs within the first 7 days following stroke and will be completed either in person or remotely to reduce the risks associated with prolonged participant-researcher contact.

The events of Visits 1-2 may require 2-3 separate sessions over multiple days to complete, depending on the patient’s status and availability. Events from Visit 1 not required for determination of eligibility may be completed by the end of Visit 2.

Enrollment is defined as the date of randomization, after all screening criteria have been met (typically immediately following the Western Aphasia Battery-R during Visit 2). Randomization, and thus enrollment, must occur within the first 7 days following stroke.

**Note: The events of Visit 2 are completed centrally by Johns Hopkins School of Medicine clinical and research team members.**

**The following procedures will be performed:**

- - - 1. Administer the *Western Aphasia Battery-Revised* (WAB-R):

The WAB-R will characterize the participants’ overall language impairment through the evaluation of the main clinical aspects of language functioning, including speech content, speech fluency, auditory comprehension, repetition, and naming. The WAB-R allows for the differentiation of these specific language abilities, as well as the classification of aphasia type. The WAB-R also yields a composite score, the Aphasia Quotient, which provides an overall measure of severity, in which lower scores denote more severe aphasia (Kertesz, 2007). AQ will be used to stratify participants for randomization. This randomization will be done using the WebDCU^TM^ (Web Data Collection Unit, after data for the participant are entered by the speech-language pathologists).

Speech-language pathologists (SLPs) will refer to the manual for explicit instructions regarding administration and scoring procedures and administrations of the WAB-R during the acute post-stroke hospitalization may be used (i.e., the WAB-R does not need to be repeated for the study if it was already completed by the SLP as part of routine care and the full scores of that administration are available when the patient is consented). Administration time will range between 30-45 minutes. **The WAB-R is only administered a single time, at the start of Visit 2, and this WAB-R administration is used to determine eligibility for participation and randomization (AQ < 93.8).**

1. Administer the *Boston Naming Test-Second Edition* (BNT):

The BNT represents a measure of object naming abilities from a corpus of 60 line drawings. Object names are ranked along a continuum, with easier, higher frequency words appearing at the beginning of the test and more difficult, lower-frequency words appearing near the end. To eliminate participant frustration, the BNT implements a ceiling effect so that once the participant incorrectly names eight items in a row, testing will cease, with the assumption that (s)he would not correctly name the upcoming, more difficult words (Kaplan, Goodglass, & Weintraub, 2001). SLPs will refer to the manual for explicit instructions regarding administration and scoring procedures. Administration time will range between 5-20 minutes.

1. Administer the modified Rankin Scale (mRS) (Banks, 2007). This scale will provide a measure of overall disability. The SLP will refer to the instructions for scoring.
2. Administer the Stroke and Aphasia Quality of Life scale (SAQOL-39; Hilari et al., 2003). This scale provides a comprehensive examination of the impact of the stroke on multiple facets of body function, activity, and participation following stroke. The SLP will refer to the instructions for scoring. This scale will be administered only to participants who score >90% correct on the WAB-R yes/no questions at baseline.
3. Administer the Positive and Negative Affect Scale (PANAS-SF) (Watson, Clark, & Tellegen, 1988). This scale provides a snapshot of an individual’s recent emotional state, an important aspect of quality of life (QOL). The SLP will refer to the instructions for scoring. This scale will be administered only to participants who score >90% correct on the WAB-R yes/no questions at baseline.
4. Administer the Multidimensional Scale of Perceived Social Support (MSPSS) (Zimet, Dahlem, Zimet, & Farley, 1988). This scale provides an inventory of an individual’s support network, and important protective factor for quality of life and positive outcomes. The SLP will refer to the instructions for scoring. This scale will be administered only to participants who score >90% correct on the WAB-R yes/no questions at baseline.
5. Administer the Brief Resilient Coping Scale (BRCS) (Sinclair & Wallston, 2004). This four-question scale examines an individual’s resiliency to difficult situations and traumatic events, an important factor for understanding QOL change due to stroke. The SLP will refer to the instructions for scoring. This scale will be administered only to participants who score >90% correct on the WAB-R yes/no questions at baseline.
6. Administer the Big Five Inventory, Second Edition (BFI-2) (Soto & John, 2017) Negative emotionality (Ne) items. This scale examines an individual’s trait-like differences in personality and temperament, factors suspected to influence an individual’s vulnerability to decreased QOL due to stroke. The SLP will refer to the instructions for scoring. This scale will be administered only to participants who score >90% correct on the WAB-R yes/no questions at baseline.
7. Administer the General Anxiety Disorder-7 (GAD-7) (Spitzer et al., 2006). Complementing the PHQ-9, this seven-item measure quickly screens for major anxiety disorders. The SLP will refer to the instructions for scoring. This scale will be administered only to participants who score >90% correct on the WAB-R yes/no questions at baseline.
8. Administer the Life Events Checklist-5 (LEC-5) (Weathers et al., 2013). This checklist examines an individual’s prior exposure to significant trauma, a factor suspected to influence an individual’s vulnerability to decreased QOL due to stroke. The SLP will refer to the instructions for scoring. This scale will be administered only to participants who score >90% correct on the WAB-R yes/no questions at baseline.

Tests 11-13 will help us identifying the underlying cause of impairments in naming and discourse

1. Administer the *Morphosyntactic Generation* (MorGen) test (Administration time will range between 15-30 minutes).
2. Administer the “Cinderella Story” picture discourse analysis.

Place the picture book in front of the participant. Tell the participant, "I'm going to ask you to tell a story. Have you ever heard the story of Cinderella?" (Make note of answer) "Do you remember much about it? These pictures might remind you of how it goes. Take a look at the pictures and then I'll put the book away and ask you to tell me the story in your own words." Allow the participant to look through the book (assist with page turning, if needed) and then, if necessary, prompt: "Now tell me as much of the story of Cinderella as you can. You can use any details you know about the story, as well as the pictures you just looked at." Continue until the participant concludes the story or it is clear s/he has finished.

1. Baseline assessment of primary outcome measure (Philadelphia Naming Test, short form)
   1. Turn on the laptop computer and position in front of the participant or allow participant to view researcher’s screen.
   2. Set up and start internal web-camera for audio-visual recording.
   3. Administer the short form of the *Philadelphia Naming Test* (PNT) on a laptop computer.
   4. Instruct the participant to overtly name each picture as soon as it is displayed. Trials will end following a response or after 20-seconds have elapsed, in which the administrator will say the correct picture name in order to discourage perseveration on subsequent trials
   5. Stop web-camera and save video file for later scoring of naming.

#### **Randomization**

The “Real-Time” randomization will take place centrally via the Trial Website. Subjects will be randomized 1:1 (placebo: escitalopram), controlling for severity (classified using the Western Aphasia Battery revised: WAB-R). The computer program developed at the DCU makes the treatment assignment based on the current status of treatment group distribution within each stratum as well as overall balance of treatment assignment.

The center staff enters the baseline AQ (severity) and eligibility information of a subject prior to randomization. If the subject’s eligibility status is confirmed, the computer program on the WebDCU^TM^ server will evaluate the treatment arm and severity distributions and assign a double-blinded randomization code based on the randomization scheme. The SLP randomizing the participant will not know the treatment assignment, only a subject identification number.

The unblinded list of randomization codes and treatment assignments will be generated by the DCU and communicated to the research pharmacies.

**Randomization will occur no later than 7 days following the initial stroke and marks the beginning of the drug trial period.**

**Following the initial onboarding visits (Visits 1-2), participants should be sent home with the following materials to complete the remainder of the study:**

- - - 1. **Study drug (or placebo)**
      2. **Blood pressure and heart rate monitor**
      3. **Materials for remote completion of the CoDeNT therapy: study laptop, earbuds/earphones, WiFi hotspot (as needed)**

**Please be sure to remind participants of weekly monitoring calls they will receive from research team members.**

### 6.2.3 Blinding

The study is to be conducted in a double-blind manner. The subjects, the site investigators, and the clinical staff involved in this study will not know the treatment assignment. The statistical team at the Statistical and Data Management Center will be unblinded. The study statistician will provide a sealed envelope with the treatment group identifiers to the DSMB. This envelope would only be opened by the DSMB if they require unblinding or at the end of the study.

### 6.2.4 Follow-up Visits

Participants will receive weekly phone calls (typically on Monday unless otherwise arranged) beginning the first Monday after randomization (post-stroke day 0-7) to administer the PHQ-9, count pills, answer questions from participants and their families, and record any adverse events. Pills will continue to be counted at each visit until the drug treatment period and taper end (post-randomization day 104).

**Visit 3 (no fewer than 14 days post-randomization + 0-7 days)**

Visit 3 is a safety monitoring visit and requires direct contact for outpatient testing.

1. Obtain electrocardiogram and email to David Spragg (JHU) for central interpretation.
2. Count pills (if at home) or review medication administration records (if hospitalized)
3. Check electrolytes (blood test).
4. Record any Adverse Events experienced during the treatment session or since the last visit on the AE case report form. If adverse events are noted, a targeted physical examination will be completed during this visit, either in person or remotely.

**Visits 4-18 (no fewer than 60 days post-randomization + 0-7 days): CoDeNT naming treatment**

Visits 4-18 (CoDeNT treatment sessions 1-15) typically occur within 3-4 weeks, beginning post-randomization day 60-67 and ending post-randomization day 81-90. Each session is approximately 45-60 minutes in length. In addition to the PHQ-9 administered weekly throughout the study, the neurological examination administered during Visit 1 will be re-administered by Dr. Hillis during the treatment phase Visits 8, 13, and 18. Pill checks will continue throughout CoDeNT treatment visits, as drug treatment is ongoing. Patients who are no longer experiencing a disruption in language skills will have the option of not completing CoDeNT but will continue to be followed throughout the remainder of the study course.

**Note: All treatment sessions must be completed by post-randomization day 90.**

The following procedures will be performed:

Procedures for Treatment

The following procedures will be performed remotely over video conferencing software by the research team facilitated by the site coordinator:

1. Set-up the computer-delivered naming task:
   - - The participant and/or their caregiver will receive instructions on how to turn on computer, use headphones, pair the hot-spot with the computer, and start the therapy program. Members of the JHU team will plan a call ahead of the start of treatment sessions to assist participants with setup as needed.
2. Guide the participant in measuring blood pressure and heart rate using the automated monitor provided by the research team. Record the results.
3. Instruct the participant how to perform the self-administered computer-delivered naming treatment, consisting of a picture/seen and heard spoken word verification task
   - - The computerized treatment task will be 45-minutes in total length
     - A picture will be presented for 2 seconds on a laptop computer screen and will be immediately followed by an audio-visual display of a male speaker’s mouth saying a noun. Video of the speaker producing the noun is presented in synchrony with the audio via in-ear headphones. The spoken word either will or will not match the preceding picture. In the event of a match, the participant indicates this response on the touch-screen using a green button. In the case of a non-match, participant is instructed to indicate this response on the touch screen using a red button. Half of the picture/word pairs will match, while the other half will not. The computer will provide immediate visual feedback following a response in the form of a “smiley face” for correct answers and a “frowny face” for incorrect answers. Additionally, following the completion of a treatment session, a data file of the participant’s responses will be automatically saved, and the accuracy score from that session will be displayed on the computer screen.


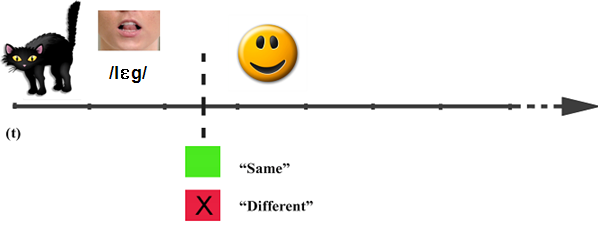


*Right: Example of the computerized treatment task. The picture representation for “cat” does not match the subsequent audio-visual presentation for “leg.” Since the pair is not a match, the red button was correctly pressed, which lead to the immediate “smiley face” feedback.*

1. Guide the participant in measuring blood pressure and heart rate using the automated monitor provided by the research team. Record the results.
2. Record any Adverse Events experienced during the treatment session or since the last visit on the AE case report form.

Visits 8, 13, and 18: Conduct neurological examination either in person or remotely via video call with a neurologist, including the NIH Stroke Scale (NIHSS) (Lyden, 1999)

- 1. Clinicians will be certified in administration of the NIHSS by taking a web-based course offered by the American Heart Association.
  2. The NIHSS will provide a measure of overall stroke severity.

**Visit 19 (7 days from the end of CoDeNT + 0-7 days AND no fewer than 93 days post-randomization +0-7 days)**

Minimum time post-randomization: 93 days

Maximum time post-randomization: 104 days

Visit 19 **requires direct contact** to complete neuroimaging, ECG, check electrolytes (blood test), and administer the grip strength and peg board tasks at each participant’s recruitment site. Contact duration may be abbreviated by dividing testing into two shorter sessions, one remote and one in person.

Pills will continue to be counted at each visit until the drug treatment period and taper end (post-randomization day 104). The events of Visit 19 may require 2-3 separate sessions over multiple days to complete, depending on the participant’s status and availability.

The following procedures will be performed:

1. Post-naming treatment assessment of primary outcome measure (Philadelphia Naming Test, short form)
   1. Turn on the laptop computer and position in front of the participant or allow participant to view researcher’s screen.
   2. Set up and start internal web-camera for audio-visual recording.
   3. Administer the short form of the *Philadelphia Naming Test* (PNT) on a laptop computer.
   4. Instruct the participant to overtly name each picture as soon as it is displayedTrials will end following a response or after 20-seconds have elapsed, in which the administrator will say the correct picture name in order to discourage perseveration on subsequent trials
   5. Stop web-camera and save video file for later scoring of naming.
2. Administer the “Cinderella Story” picture discourse analysis.

Place the picture book in front of the participant. Tell the participant, "I'm going to ask you to tell a story. Have you ever heard the story of Cinderella?" (Make note of answer) "Do you remember much about it? These pictures might remind you of how it goes. Take a look at the pictures and then I'll put the book away and ask you to tell me the story in your own words." Allow the participant to look through the book (assist with page turning, if needed) and then, if necessary, prompt: "Now tell me as much of the story of Cinderella as you can. You can use any details you know about the story, as well as the pictures you just looked at." Continue until the participant concludes the story or it is clear s/he has finished.

1. Administer the modified Rankin Scale (mRS) (Banks, 2007). This scale will provide a measure of overall disability. The SLP will refer to the instructions for scoring.
2. Administer the *Morphosyntactic Generation* (MorGen) test (Administration time will range between 15-30 minutes).
3. Administer the Stroke and Aphasia Quality of Life scale (SAQOL-39). This scale will be administered only to participants who score >90% correct on the WAB-R yes/no questions at baseline.
4. Administer the Positive and Negative Affect Scale (PANAS-SF). This scale will be administered only to participants who score >90% correct on the WAB-R yes/no questions at baseline.
5. Record any Adverse Events experienced during the treatment session or since the last visit on the AE log. If adverse events are noted, a targeted physical examination will be completed during this visit, either in person or remotely.

**A portion of this visit must be completed in person by the participant’s recruitment site:**

1. Conduct functional imaging baseline evaluation. This will include either MRI or fNIRS.

- 1. Structural and rsfMRI will be acquired in the hospital.
     1. Run the participant on a 10-minute resting state functional connectivity MRI exam during visits two and immediately after treatment periods 1 and 2:
     2. Instruct the participant to lie still in the scanner, eyes open
     3. Run the participant on high-resolution anatomical MRI scans during each of the same visits
     4. Instruct the participant to lie still during these scans
  2. fNIRS will be conducted in a clinical space (i.e., hospital or outpatient clinic).

1. Grip strength and 9-peg board tests
2. Obtain electrocardiogram or collect results from hospitalization record and email to David Spragg (JHU) for central interpretation.
3. Check electrolytes (blood test) or collect results from hospitalization record

**Visit 20 (35 days from the end of CoDeNT + 0-7 days)**

Minimum time post-randomization: 116 days

Maximum time post-randomization: 132 days

Visit 20 may be completed either in person or remotely to reduce risks associated with participant-researcher contact. The following procedures will be performed. The events of Visit 20 may require 2-3 separate sessions over multiple days to complete, depending on the participant’s status and availability.

1. Post-naming treatment assessment of primary outcome measure (Philadelphia Naming Test, short form)
   1. Turn on the laptop computer and position in front of the participant or allow participant to view researcher’s screen.
   2. Set up and start internal web-camera for audio-visual recording.
   3. Administer the short form of the *Philadelphia Naming Test* (PNT) on a laptop computer.
   4. Instruct the participant to overtly name each picture as soon as it is displayed. Trials will end following a response or after 20-seconds have elapsed, in which the administrator will say the correct picture name in order to discourage perseveration on subsequent trials
   5. Stop web-camera and save video file for later scoring of naming.
2. Administer the “Cinderella Story” picture discourse analysis.

Place the picture book in front of the participant. Tell the participant, "I'm going to ask you to tell a story. Have you ever heard the story of Cinderella?" (Make note of answer) "Do you remember much about it? These pictures might remind you of how it goes. Take a look at the pictures and then I'll put the book away and ask you to tell me the story in your own words." Allow the participant to look through the book (assist with page turning, if needed) and then, if necessary, prompt: "Now tell me as much of the story of Cinderella as you can. You can use any details you know about the story, as well as the pictures you just looked at." Continue until the participant concludes the story or it is clear s/he has finished.

1. Administer the modified Rankin Scale (mRS) (Banks, 2007). This scale will provide a measure of overall disability. The SLP will refer to the instructions for scoring.
2. Administer the Stroke and Aphasia Quality of Life scale (SAQOL-39). This scale will be administered only to participants who score >90% correct on the WAB-R yes/no questions at baseline.
3. Administer the Positive and Negative Affect Scale (PANAS-SF). This scale will be administered only to participants who score >90% correct on the WAB-R yes/no questions at baseline.
4. Conduct neurological examination either in person or remotely via video call with a neurologist, including the NIH Stroke Scale (NIHSS) (Lyden, 1999)
   1. Clinicians will be certified in administration of the NIHSS by taking a web-based course offered by the American Heart Association.
   2. The NIHSS will provide a measure of overall stroke severity. The SLP will also record the description of the “Cookie Theft” picture on the NIHSS, which will also be analyzed for Content Units (CU) and Syllable/CU (Yorkston & Beukelman, 1980).
5. Record any Adverse Events experienced during the treatment session or since the last visit on the AE log. If adverse events are noted, a targeted physical examination will be completed during this visit, either in person or remotely.

### 6.2.5 Completion/Final Evaluation

**Visit 21 (140 days from the end of CoDeNT + 0-7 days)**

Minimum time post-randomization: 221 days

Maximum time post-randomization: 237 days

Visit 21 may be completed either in person or remotely to reduce risks associated with participant-researcher contact. The following procedures will be performed. The events of Visit 21 may require 2-3 separate sessions over multiple days to complete, depending on the participant’s status and availability.

1. Post-naming treatment assessment of primary outcome measure (Philadelphia Naming Test, short form)
   1. Turn on the laptop computer and position in front of the participant or allow participant to view researcher’s screen.
   2. Set up and start internal web-camera for audio-visual recording.
   3. Administer the short form of the *Philadelphia Naming Test* (PNT) on a laptop computer.
   4. Instruct the participant to overtly name each picture as soon as it is displayedTrials will end following a response or after 20-seconds have elapsed, in which the administrator will say the correct picture name in order to discourage perseveration on subsequent trials
   5. Stop web-camera and save video file for later scoring of naming.
2. Administer the “Cinderella Story” picture discourse analysis.

Place the picture book in front of the participant. Tell the participant, "I'm going to ask you to tell a story. Have you ever heard the story of Cinderella?" (Make note of answer) "Do you remember much about it? These pictures might remind you of how it goes. Take a look at the pictures and then I'll put the book away and ask you to tell me the story in your own words." Allow the participant to look through the book (assist with page turning, if needed) and then, if necessary, prompt: "Now tell me as much of the story of Cinderella as you can. You can use any details you know about the story, as well as the pictures you just looked at." Continue until the participant concludes the story or it is clear s/he has finished.

1. Administer the modified Rankin Scale (mRS) (Banks, 2007). This scale will provide a measure of overall disability. The SLP will refer to the instructions for scoring.
2. Administer the Stroke and Aphasia Quality of Life scale (SAQOL-39). This scale will be administered only to participants who score >90% correct on the WAB-R yes/no questions at baseline.
3. Administer the Positive and Negative Affect Scale (PANAS-SF). This scale will be administered only to participants who score >90% correct on the WAB-R yes/no questions at baseline.
4. Administer the Multidimensional Scale of Perceived Social Support (MSPSS) (Zimet, Dahlem, Zimet, & Farley, 1988). This scale will be administered only to participants who score >90% correct on the WAB-R yes/no questions at baseline.

1. Administer the Life Events Checklist-5 (LEC-5). This scale will be administered only to participants who score >90% correct on the WAB-R yes/no questions at baseline.
2. Conduct neurological examination either in person or remotely via video call with a neurologist, including the NIH Stroke Scale (NIHSS) (Lyden, 1999)
   1. The SLP will also record the description of the “Cookie Theft” picture on the NIHSS, which will also be analyzed for Content Units (CU) and Syllable/CU (Yorkston & Beukelman, 1980).
3. Record any Adverse Events experienced during the treatment session or since the last visit on the AE log. If adverse events are noted, a targeted physical examination will be completed during this visit, either in person or remotely.

Procedure for Monitoring for Depression

Every Monday during the drug treatment phase of the study (post-randomization day 0-104), a speech language pathologist (SLP) or physician study team member will assess and record the participant's score on the PHQ-9, either in person or by telephone. If the participant is unable to understand the questions, the caregiver will be asked about vegetative symptoms of depression (insomnia or hypersomnia, anorexia, apathy). However, only a score of >15 on the PHQ-9 or expressed suicidal ideation by a person able to comprehend the PHQ-9 or convey suicidal ideation will be a reason for withdrawing the person from the study and providing an informed referral to the site psychiatrist. Comprehension will be assessed by asking the first 3 questions with different (synonymic) words or sentence structure to determine the reliability of the responses. 100% agreement in yes/no responses will be considered reliable. Reliability assurance will be recorded with WebDCU^TM^ and only reliable scores will be recorded to be used as independent variables in the analyses.

**Research team members will refer to the Suicidality Risk Minimization Plan, which outlines protocols for responding to PHQ-9 responses endorsing suicidality throughout the study term.**

During these calls, the study team member will record pill counts and record any Adverse Events experienced during the treatment session or since the last visit on the AE case report form.

Enhanced participant risk reduction contingency

In the event of pandemic, direct participant contact will be minimized in the protocol wherever possible. “Visits” will be divided into remote sessions with participants, which will be facilitated by video conferencing software deemed sufficiently private and confidential by IRB and abbreviated in person interactions.

Beyond this contingency, researchers and participants will engage in the following risk reduction practices prior to and during participant contact in the event of pandemic or other global public health concerns:

- Research team members will reference each university’s policies for self-screening and will defer any in-person activities if they suspect that they have any active COVID-19 symptoms.
- Within one week of scheduled in-person visit, the participant will be screened for COVID-19 symptoms. Screening will then be repeated on the testing day. Screening will include questions about other members of the participant’s household experiencing symptoms. If symptoms are reported, the participant’s visit will be cancelled.
- Researchers will practice safer-contact procedures during in-person visits. Researchers will arrive wearing cloth masks and face shields and retain them in all in-person encounters with participants. Participants will be required to wear a face mask for the duration of each in-person visit. If the participant does not have a mask, one will be provided for the duration of testing.
- Researchers will practice hand hygiene and use gloves when in contact with participants. Equipment and work surfaces will be disinfected prior to and following each session.

The above risk reduction practices are implied as necessary during all visits where in-person procedures are described.

# 7. SAFETY ASSESSMENTS

Potential adverse experiences by intervention (alphabetical):

Escitalopram

- *Adverse events following abrupt discontinuation of escitalopram:* Escitalopram has been associated with spontaneous reports of adverse events occurring upon discontinuation, particularly when abrupt, including the following:
  - Agitation
  - Anxiety
  - Confusion
  - Dizziness
  - Dysphoric mood
  - Emotional lability
  - Headache
  - Hypomania
  - Insomnia
  - Irritability
  - Lethargy
  - Sensory disturbances (e.g., paresthesias such as electric shock sensations)

**Mitigation:** Although these events are associated with discontinuation of escitalopram used in the treatment of underlying psychiatric illness, participants will be tapered off the medication by taking 5 mg escitalopram (or placebo) for 2 weeks after the 90 days of treatment to mitigate the risk of these events.

- *Hyponatremia:* Hyponatremia can occur as a result of treatment with escitalopram, in many cases due to the development of the syndrome of inappropriate antidiuretic hormone secretion (SIADH).

**Mitigation:** Participants receive bloodwork monitoring for sodium before beginning escitalopram and during the first post-treatment evaluation (Visit 3). We will discontinue drug treatment in participants who develop serious hyponatremia (Na < 130), but these participants will continue to be followed through the end of the study term.

- *QT prolongation*: An uncommon, but more serious side effect is prolongation of the QT interval (QTc) on electrocardiogram, which can lead to a hazardous arrhythmia. In healthy individuals, the mean QTc is approximately 400 ms (up to 450 ms for men and 460 ms for women). A QTc longer than 450 ms or an increase in QTc greater than 60 ms is considered a major risk factor for dangerous arrhythmia. (Lam, 2013).

**Mitigation:** Participants receive electrocardiograms before beginning escitalopram and during the first post-treatment evaluation (1 week post-treatment). If participants demonstrate a QTc greater than 450 milliseconds on the pre-treatment electrocardiogram they will be excluded from the study. Those who develop a QTc greater than 450 during any of the visits will be removed from the study drug condition but will continue to be followed through the end of the study term.

- *Suicidality*: Increased risk of suicidal thinking and behavior (suicidality) in children, adolescents, and young adults in short-term studies of major depressive disorder (MDD) and other psychiatric disorders. However, there was a reduction in risk with antidepressants compared to placebo in adults aged 65 and older.

**Mitigation:** Participants with antidepressant use at the time of stroke onset or moderately severe depression (PHQ-9 score >15) at enrollment are excluded from the present study to minimize the possibility that individuals with MDD will be enrolled in the study. Participants who score >15 on the PHQ-9 or express suicidality will be seen by the study psychiatrist and tapered off medication/placebo or receive other intervention recommended by the psychiatrist. Refer to the **Suicidality Risk Minimization Plan** for additional details.

- Side effects also common with placebo:
  - Constipation
  - Drowsiness
  - Dry mouth
  - Insomnia
  - Nausea

**Mitigation:** Medication will be up-titrated by taking 5 mg escitalopram (or placebo) for the first week, as this has been noted to decrease likelihood of common, mild somatic side effects.

MRI

The present study involves that participants undergo MRI scanning. The effects of undergoing MR scanning have been extensively studied and there are no known risks associated with an MR exam. The participant may, however, be bothered by:

- *Claustrophobia* associated with entering the scanner

**Mitigation:** All participants will have a “panic button” to request an end of the study if they feel uncomfortable while in the scanner. Microphone communication, audio, and video of the scanner area are standard monitoring features in all MRI scanner suites to assist in identifying and responding to patients in distress.

- *Incidental findings:* There is a psychological risk of identifying unexpected findings on brain MRI (aneurysm, tumor, etc.). This risk will be very low, as all participants will have had previous brain imaging including MRI (if they have no contraindication for MRI) as part of their stroke evaluation.

**Mitigation:** If an incidental finding is observed, the participant will be asked permission to contact the primary care physician about the abnormality and will be offered a timely appointment with a neurologist, neurosurgeon, or other physician if appropriate.

- *Noise* made by the magnet during the procedure

**Mitigation:** Participants will be asked to wear earplugs or earphones while in the magnet.

## Specification of Safety Parameters

The participant may stop testing or the intervention any time. There will be emergency personnel and equipment on hand for safety during in-person visits. QTc will be evaluated with electrocardiogram (ECG), reviewed by the consulting cardiologist at JHMI (Dr. Spragg), before starting escitalopram (Visit 1), during the drug treatment period (Visit 3), and approximately one week following the end of CoDeNT (Visit 19). ECGs obtained at each site will be scanned and sent to Dr. Spragg securely to review. If an unexpected finding is identified, Dr. Spragg will ask for permission to contact the primary care physician and will arrange a prompt evaluation by a cardiologist or other appropriate physician. Sodium and other electrolytes will be evaluated at the same visits. If participants have a prolonged QTc or significant hyponatremia (Na<130), they will be removed from the study drug treatment but will continue to be followed through the end of the study term.

If an unexpected finding is identified on MRI, the study physician (Dr. Hillis or the site PI) will explain the finding and will ask permission to contact the primary care physician and will arrange prompt evaluation by a neurologist, neurosurgeon, or other appropriate physician.

## Methods and Timing for Assessing, Recording, and Analyzing Safety Parameters

Adverse events will be monitored during the entire visit by the study team. The families will be given telephone numbers of study team as well. The site PI and the DSMB will be notified immediately if any serious adverse events are reported. DSMB members will review SAEs within 24 hours of when the PI or other study team member becomes aware of the SAE and will determine if the SAE is related to the study, and what actions (if any) are required in response to the SAE. If a significant safety concern arises, participants may be unblinded in order to address it. Adverse events will be monitored until they are resolved or clearly determined to be due to a subject’s stable or chronic condition or intercurrent illness. Medical care will be provided, as defined in the informed consent, for any adverse event related to trial participation. Appropriate medical care will include initiating transport to the Emergency Department for evaluation when necessary. All adverse events, regardless of intensity or causality, will are to be recorded in the study documentation and reported to the JHU IRB and DSMB. Any serious adverse events will be reported to the IRB and the DSMB within 24 hours.

All adverse experiences will be summarized in terms of frequency, severity and relatedness to the study treatment using the MedDRA code. All subjects who received escitalopram will be included in the safety analysis. At the end of the study, the cumulative incidences of adverse events are compared between the two treatment groups using Fisher’s exact test at the two-sided alpha level of 0.05.

## Adverse Events and Serious Adverse Events

An **adverse event (AE)** is defined as any unfavorable and unintended diagnosis, symptom, sign (including an abnormal laboratory finding), syndrome or disease which either occurs during the study, having been absent at baseline, or if present at baseline, appears to worsen.

A **serious adverse event (SAE)** is any untoward medical occurrence that results in death, is life threatening, requires inpatient hospitalization or prolongation of existing hospitalization, results in persistent or significant disability/incapacity, or is a congenital anomaly.

Electrocardiograms and blood tests will be collected to assess safety. Thresholds for exclusion at the pre-treatment evaluation or removal from the drug treatment at the mid-treatment visit are:

- Hyponatremia (Na < 130)
- QTc greater than 450 milliseconds

No AE will be specifically solicited beyond the evaluations before and immediately following treatment. Unsolicited events will be monitored during all visits as reported by participants and captured in the AE case report form.

## 7.4 Reporting Procedures

The study biostatistician will generate closed and open DSMB reports semi-annually or more frequently, as determined by the DSMB. Each DSMB report provides cumulative summary statistics on enrollment; subject status in the study (e.g., number completed study, dropouts, etc.); baseline characteristics; safety data, including AEs and SAEs; and data quality information. The statistics for the closed DSMB Reports are provided by treatment group displayed as A or B. The open report contains aggregated statistics only, i.e., not by treatment group.

## 7.5 Follow-up for Adverse Events

Adverse events will be monitored until they are resolved or clearly determined to be due to a subject’s stable or chronic condition or intercurrent illness. Medical care will be provided, as defined in the informed consent, for any adverse event related to trial participation. Appropriate medical care will include initiating transport to the Emergency Department for evaluation when necessary.

## 7.6 Safety Monitoring

The study biostatistician will generate closed and open DSMB reports semi-annually or more frequently, as determined by the DSMB. Each DSMB report provides cumulative summary statistics on enrollment; subject status in the study (e.g., number completed study, dropouts, etc.); baseline characteristics; safety data, including AEs and SAEs; and data quality information. The statistics for the closed DSMB Reports are provided by treatment group displayed as A or B. The open report contains aggregated statistics only, i.e., not by treatment group. The DSMB will monitor safety at least semi-annually and decide if the study should continue or be terminated early.

# 8. INTERVENTION DISCONTINUATION

Intervention for a participant will be discontinued if any of the following criteria are met:

- The participant requests removal from the study
- The participant exhibits high suicidality/severe depression (PHQ > 15)
- The participant exhibits hyponatremia (Na < 130)
- The participant exhibits a QTc greater than 450 milliseconds
- The participant is diagnosed with schizophrenia or another psychiatric or neurological condition that affects naming/language (self-report)
- The participant is diagnosed with a psychiatric condition requiring interventions prohibited while on escitalopram (e.g., severe depression, bipolar disorder, by self-report; see 5.3.3).
- The participant experiences sudden visual or hearing loss (self-report)
- The participant becomes pregnant during the drug treatment (self-report)
- The participant starts a medication that is contraindicated (self-report, see 5.3.3)

As long as it is not medically inadvisable, participants whose treatment is discontinued during the drug treatment term will complete a 5 mg taper off of escitalopram for 2 weeks in order to mitigate the risk for side-effects due to discontinuation of the drug. No additional evaluations will be conducted following discontinuation.

The study may be discontinued at any time by the IRB, the NIDCD, the OHRP, the FDA, or other government agencies as part of their duties to ensure that research participants are protected. The study may be discontinued if the study is closed by Johns Hopkins School of Medicine or C-STAR.

# 9. STATISTICAL CONSIDERATIONS

## 9.1 General Design Issues

The primary hypothesis is H_0_: mu_1_<mu_2_ versus H_A_: mu_1_> mu_2_, where mu_1_ is the mean change in accuracy of naming untrained items between baseline and 1 week post-CoDeNT (Visit 19, 93-104 days post-randomization) in the escitalopram group and mu_2_ is the mean change in accuracy of naming untrained items between baseline and 1 week post-CoDeNT in the placebo group.

Hypothesis 1.b. The effect of escitalopram on language improvement is independent of its effect on depression.

Hypothesis 1.c. The effect of escitalopram relative to placebo is significantly greater in individuals with infarcts involving left superior temporal gyrus (STG) and/or arcuate fasciculus (AF), compared to individuals without damage to left STG or AF.

Hypothesis 2.a. Greater improvement in language is associated with increased functional connectivity within the left hemisphere language network as measured by rsfMRI or fNIRS in participants who receive escitalopram, but not in those who receive placebo, independently of improvement in depression.

Hypothesis 2.b. Greater improvement in depression is associated with changes in connectivity in frontolimbic circuits in participants who received escitalopram, but not in those who receive placebo, independently of improvement in language. The goal of this exploratory aim is to determine whether or not we will be able to separate the effects of escitalopram on language and depression, using changes in network connectivity.

Hypothesis 2.c. Individuals with met alleles of BDNF show reduced response to treatment and reduced changes in connectivity in the language network, compared to those with the normal val/val alleles.

Secondary Analyses

In addition to the primary outcome, several secondary analyses will be conducted. We will examine changes in types of naming errors (defined by the PNT) by treatment group. The Cinderella story will be analyzed by comparing:

1) Lexical diversity (VOCD) for nouns, verbs and adjectives

2) Number and types of errors

3) Length and patterns of pauses by treatment group.

Pre and post comparisons with both non-aphasic and aphasic speakers from the Aphasia Bank database who share a number of demographic features (e.g., type and severity of aphasia, age, etc.) will also be made. Secondary outcomes also will include changes in performance by treatment group on the Content Units (CU) and syllables/CU in describing the Cookie Theft picture on the NIH Stroke Scale, percent correct morphosyntactic production on the MorGen, peg board, and grip strength.

Exploratory examinations further will evaluate differences between treatment groups in dimensions of quality of life as measured by:

1) mRS

2) Stroke Aphasia Quality of Life scale (SAQOL-39)*

3) Positive and Negative Affect Scale (PANAS-SF)*

4) Multidimensional Scale of Perceived Social Support (MSPSS)*

5) Brief Resilient Coping Scale (BRCS)*

6) Big Five Inventory (BFI-2) negative emotionality items*

7) Life Events Checklist-5 (LEC-5)*

*These inventories will be administered only to participants who score >90% correct on the WAB-R yes/no questions at baseline.

At the end of the study, for the interval scale variables, mean change from baseline to immediate post-testing in secondary outcome measures will be reported by treatment group along with the 95% confidence intervals. Treatment comparisons will be made with a paired t-test. For binary variables, the proportion of subjects immediately post-testing will be reported by treatment group along with the 95% confidence intervals.

Post-Testing Phase

The longer follow-up post-testing phase will provide exploratory information on whether the immediate post-testing improvement after 3 weeks of treatment can be sustained. The mean (95% CI) changes from baseline to immediate (within 1 week) post-CoDeNT testing, 5 weeks post-CoDeNT testing and 20 weeks post-CoDeNT testing will be reported. Box and whisker plots will be produced to show the distribution of naming accuracy over time by treatment group. To explore the longitudinal data, a general linear mixed model (GLMM) will be constructed by treatment group for the dependent variable (naming accuracy). The GLMM will incorporate random subject effects to account for repeated measurements being made on subjects; the model will include time, aphasia type and severity, number of SLP treatment sessions other than CoDeNT, PHQ-9 score, and clinical center as independent variables. A similar modeling approach may be applied to other secondary outcomes.

Safety Analyses

All adverse events will be summarized in terms of frequency, severity and relatedness to the study treatment using the MedDRA code. All subjects who received escitalopram will be included in the safety analysis. At the end of the study, the cumulative incidences of adverse events are compared between the two treatment groups using Fisher’s exact test at the two-sided alpha level of 0.05.

## Sample Size and Randomization

We expect to enroll 88 participants over 4 years (allowing 6 months to begin recruitment and 6 months for follow up at the end of the study) and expect at least 56 will complete the study on the study drug. However, all participants randomized will be included in the intent-to-treat analysis. We predict no difficulty recruiting at least 22 people with aphasia due to acute stroke each year. The PI has recruited an average of 13 people with aphasia due to acute left hemisphere ischemic stroke each year in her ongoing treatment study with tDCS at JHH and JHBMC, with similar inclusion and exclusion criteria. We anticipate having more difficulty enrolling in this trial, because of the exclusion criteria of moderately-severe depression or use of SSRIs at the time of stroke. We also plan for a relatively high dropout rate/crossovers, because of the risk of developing moderate-severe depression or other events for which stroke patients are at risk (e.g. recurrent stroke) and a small risk of developing prolonged QTc interval on electrocardiogram (see Human Subjects section) on escitalopram. If a sample size in each group is of 28 per group (N = 56 total), we will have 90% power to detect a Cohen’s d effect size of 0.8 using a two group t-test with a one-sided alpha of 0.05. We observed Cohen’s d effect sizes larger than 1 in improvements in naming. These mean changes for active and placebo groups and standard deviation of change are based on the changes in accuracy in amongst naming in people with aphasia who took SSRIs for 3 months and versus those who took no SSRIs in our recently published longitudinal observational study; however, since these effects were based upon small samples, we have conservatively powered this study for a smaller effect than previously observed. Since this is a phase II trial, the type I error rate has been relaxed to 0.05 one-sided, but we have maintained a high power (90%). If the true effect size is smaller than expected, we still have adequate power.

### 9.3 Treatment Assignment Procedures

The randomization will take place centrally via the Trial Website. Subjects will be randomized 1:1 (placebo: escitalopram), controlling for severity (classified using the Western Aphasia Battery revised: WAB-R). The computer program developed at the DCU makes the treatment assignment based on the current status of treatment group distribution within each stratum as well as overall balance of treatment assignment.

A “Real-Time” randomization procedure is implemented via the Trial Website on the WebDCU^TM^.

The center staff enters the baseline AQ (severity) and eligibility information of a subject prior to enrollment. If the subject’s eligibility status is confirmed, the computer program on the WebDCU^TM^ server will evaluate the treatment arm distribution and generate a blinded treatment assignment based on the randomization scheme. The SLP randomizing the participant will not see the treatment assignment, only a numeric participant number. The unblinded list of randomization codes and treatment assignments will be generated by the DCU and communicated to the research pharmacies.

Planned breaking of randomization codes: at the time of study termination.

Unplanned breaking of randomization codes: at the time of any SAE requiring a participant discontinue the study, if unblinding is required for participant safety. In this case, the site PI will utilize the WebDCU^TM^ interface to unblind what the treatment condition was, and the site PI will contact the participant’s treating physician.

*Procedure for retaining the blind*. Should data collected in the study offer evidence of a participant’s assignment to a particular study arm, such as a reduction in sodium or increase in the QTc interval, but not to the level requiring discontinuation of the study in that individual), the data will not be shared with either the participant or the SLP providing treatment or evaluation.

Individual authorized to break the blind. The site PI or member of the DSMB

- Circumstances for breaking the blind. Any SAE requiring a participant discontinue the study, if unblinding is required for participant safety. In this case, the PI will contact the WebDCU^TM^, and the staff from the WebDCU^TM^ will contact the participant’s treating physician.
- Procedure for breaking the blind. The site PI will utilize the WebDCU^TM^ interface to unblind what the treatment condition was, and the site PI will contact the participant’s treating physician.

Randomization will be stratified by severity (WAB-R AQ). There are no sample size goals for each stratum.

**Definition of Populations**

Analyses will all be based on ITT (Intent to treat) principle, based on the group to which the participant is randomized, whether or not the participant completes the treatment as planned.

## 9.4 Interim Analyses and Stopping Rules

No formal interim analyses are planned. If the DSMB determines that there is a safety concern, or the PI or IRB identifies a safety concern, the study will be stopped.

## 9.5 Outcomes

### 9.5.1 Primary Outcome

The primary hypothesis is H_0_: mu_1_<mu_2_ versus H_A_: mu_1_> mu_2_, where mu_1_ is the mean change in accuracy of naming untrained items between baseline and 1 week post-CoDeNT (Visit 19, 93-104 days post-randomization) in the escitalopram group and mu_2_ is the mean change in accuracy of naming untrained items between baseline and 1 week post-CoDeNT in the placebo group.

The primary analysis will compare change in accuracy of naming untrained items between groups (active versus placebo) in a two-sample *t*-test for the Intent-to-treat sample. For people with aphasia who do not complete the 1 week post-treatment assessment, the post-treatment value will be imputed using a multiple imputation approach assuming a monotone missing mechanism and missing is at random (MAR). As a sensitivity analysis, the primary outcome analysis will be repeated using all available follow-up data (without explicit imputation) in a mixed effects model of the change from baseline in naming accuracy adjusted for baseline (with week 1, 5, or 20 as a categorical variable and a random effect for subject). Similar analyses will be done for the change in accuracy of trained items.

### 9.5.2 Secondary Outcomes

Several secondary analyses will be conducted. We will examine changes in types of naming errors (defined by the PNT) by treatment group. The Cinderella story will be analyzed by comparing:

1) Lexical diversity (VOCD) for nouns, verbs and adjectives

2) Number and types of errors

3) Length and patterns of pauses by treatment group.

Pre and post comparisons with both non-aphasic and aphasic speakers from the Aphasia Bank database who share a number of demographic features (e.g., type and severity of aphasia, age, etc.) will also be made. Secondary outcomes also will include changes in performance by treatment group on the Content Units (CU) and syllables/CU in describing the Cookie Theft picture on the NIH Stroke Scale, percent correct morphosyntactic production on the MorGen, peg board, and grip strength.

Secondary analyses will also examine if any effects of escitalopram vary across participants with different characteristics such as age, education, presence of infarct in left superior temporal gyrus/ arcuate fasciculus (STG/AF), and lesion volume. If there is a difference between groups on any of these measures at baseline, these will be entered as regressors, along with age, education, and baseline score on the same test, in identifying variables that influenced change in primary and secondary outcome variables. A regression of change in naming accuracy, which includes main effects and interaction terms with treatment group for these characteristics will be used. This model will be adjusted for treatment and time as described above for the mixed effects model. Given the small sample size and since this is a secondary analysis, a significance level of 0.10 will be used to retain main effects or interaction terms with treatment in the final reported model.

## 9.6 Data Analyses

Multiplicity

Since this is a phase II study, the false positive error rate has been relaxed (Schoenfeld 1980). For secondary outcomes and safety analyses, no adjustment of Type I error probability will be considered, since they will be treated as exploratory.

Missing Data

Under the ITT principle, all patients who are randomized are included in the analysis. Therefore, missing data, especially in the primary outcome measure, can be problematic. For the primary futility analysis we will impute missing data using multiple imputation (Rubin, 1987) assuming a

monotone missing mechanism and missing is at random (MAR). Similar methods will be employed for secondary analyses and secondary outcomes. For safety data (e.g. AEs) no data imputation will be done.

Adjusting for Covariates

As a sensitivity analysis, the primary analysis will be repeated adjusting for enrolling site and for the duration/dose of speech, occupational, and physical therapy. Similarly, the primary outcome analysis will be repeated using all available follow-up data in a mixed effects model of the change from baseline in naming accuracy adjusted for baseline as a categorical variable and a random effect for subject. Similar analyses will be done for the change in accuracy of trained items.

# 10. DATA COLLECTION AND QUALITY ASSURANCE

## 10.1 Data Collection Forms

A blinded speech-language pathologist (SLP) to be named at each site will perform baseline and outcome assessments. Codes without any protected health information will be used for each participant Demographic and assessment data will be recorded directly on the CRFs, and this will be considered source data. The CRFs will be kept in locked cabinets, and data will be entered by another to-be-named SLP into WebDCU^TM^.

## 10.2 Data Management

Assessment and treatment apps have been developed so that video recordings of assessment and treatment tasks are uploaded automatically to HIPAA-compliant MyCloud accounts as soon as they are administered. After assessments are scored by raters, these data are entered, monitored and analyzed in WebDCU^TM^ at the Medical University of South Carolina.

## 10.3 Quality Assurance

### 10.3.1 Training

The Clinical Core is responsible for training all personnel who have contact with participants and/or friends and family members to ensure that all exchanges are ethical, respectful, and employ successful communication strategies. This includes training for communication during initial recruitment and participant identification, for the informed consent process, and for participant retention. It also includes training of assessment administration procedures and treatment procedures. This training helps to reduce variance introduced by clinicians communicating or carrying out procedures with different styles than others. Training of personnel in this manner has rarely been reported in the aphasia treatment literature.

The Clinical Core ensures that all study personnel having any contact with participants and/or the friends and family members of the participants have a basic understanding of the communication needs and obstacles for this population, and also some understanding of their experience. While most study personnel have had a background with adult neurogenic communication disorders, standardizing this baseline training for all personnel, from those who might only be escorting them to the elevator to those who spend hours with them in treatment sessions, ensures that no erroneous assumptions about qualifications are made. This baseline training comprises many components that are already implemented by the Clinical Core of C-STAR, with positive feedback and results. The Clinical Core continues to facilitate the training and maintain compliance logs.

First, all study personnel complete human subjects training (CITI) to understand ethical treatment of human research participants, which is essential training even in the earliest stages of a study (e.g., to avoid coercion and/or bias during advertising and recruitment efforts). Second, all personnel independently read two articles by the Hilari research group discussing the impact of aphasia: 1) *“Aphasia blog talk: How does stroke and aphasia affect a person’s social relationships?”* (Fotiadou, Northcott, Chatzidaki, & Hilari, 2014); and 2) *“Aphasia blog talk: How does stroke and aphasia affect the carer and their relationship with the person with aphasia.”* (Winkler, Bedford, Northcott, & Hilari, 2014). Third, all personnel independently view four videos available via AphasiaBank (<http://talkbank.org/AphasiaBank/>; participants consented to be videotaped by AphasiaBank) during which persons with aphasia discuss their stroke story. Two videotaped persons have nonfluent aphasia (1 high, 1 low severity) and two have fluent aphasia (1 high, 1 low severity). Finally, the Clinical Core leads small group sessions that include a brief lecture on *“How to communicate with individuals with aphasia”,* a lecture used by both Fridriksson and Spell when training speech-language pathology students to be aphasia group leaders. This lecture is followed by an experiential learning session utilizing the program *“Experiencing Aphasia: An Inservice Model Demonstrating Aspects of Aphasia.”*  The stated goals of the program are: (1) To give participants an opportunity to experience what it is like to be communicatively impaired; (2) To encourage participants to discuss their emotional responses to being treated as an impaired individual; and (3) To instruct participants about the different modalities that may be impaired as a result of stroke or head injury. This session will provide opportunities for personnel to participate in simulations of communicating with aphasia through receptive language, expressive language, reading, and writing stations.

**Assessment**

Borrowing from treatment fidelity guidelines, the Clinical Core will coordinate assessor and rater training, assessment delivery, and reliability of raters.

Assessors are speech-language pathologists who already have a basic understanding of the project (through baseline and informed consent training, some of whom already have received the detailed training described below, facilitated by the current Clinical Core. New assessors will complete the same training. Illustrating the relationships between the assessments the clinicians are giving and the specific project goals emphasize the importance of the assessment process and adherence to prescribed procedures. This development of clinician “meta-competence” or “buy-in” is thought to be important in treatment fidelity and is intuitive in assessment as well – understanding the “why” facilitates investment in the “how”, or adherence to procedures. In addition, highlighting the opportunities most susceptible to drift or contamination serves to bring the information to the forefront so that it can be actively avoided. Finally, clinician attrition is likely to be observed less in clinicians who are invested in the project goals and feel as if their contribution is important.

The Clinical Core will facilitate the following assessment training procedures: (1) independent reading of the assessment manual; (2) video observation of expert administration of each test, administered to persons with varying types and severity of aphasia; (3) a small group training session that includes manual review, highlighting of similarities and differences between administration procedures via discussion and video observation, and supervised role-play with feedback; (4) at project initiation, three supervised assessment sessions with expert feedback; and (5) throughout study, yearly “booster” small group training and 1-2 supervised assessment sessions with expert feedback. These training procedures guard against clinician-to-clinician variability, drift, and contamination. Both new and current assessors will receive specialized training in administration of the Morphosyntactic Generation (MorGen) test.

To continue training, SLPs meet monthly (or more often) to discuss questions about protocols. They also participate in online webinars and other professional development activities related to aphasia and apraxia.

**Treatment**

The Clinical Core optimizes treatment fidelity by monitoring multiple components, specifically clinician training, treatment delivery, and treatment receipt. This is an ongoing process, not only occurring at the onset of the project, but rather continually performed over the entire duration of the project to ensure that treatment implementation on the first day of the first year of the project is the same as on the last day of the last year of the project. As with the assessment fidelity plan, when an SLP deviates from a treatment protocol, she or he is given feedback immediately.

### 10.3.2 Quality Control Committee

The assumption of the investigators is that assessors, raters, and clinicians adhere to well-defined study protocols. The Clinical Core exists to make that a reality. By doing so, the Clinical Core guards against threats to statistical conclusion validity and internal validity, increasing the power to detect effects and increasing investigator and consumer confidence in the results. By reducing the chances of making Type I, II, or III errors, we would decrease the probability that this trial would result in research waste, the costs of which can only begin to be fathomed if one recognizes that the impact of those errors would not only be felt in this study, but throughout the entire research community. This responsibility is not taken lightly and is why the Clinical Core is essential to the research activities of C-STAR.

Overall management of the Clinical Core is in the hand of Dr. Hillis. She is assisted by Drs. Fridriksson (USC), Spell (USC), Bonilha (MUSC), and Shafie/Yu (UCI). The Clinical Core Coordinator manages and implements day-to-day operations, as described above, with ad hoc supervision by Hillis. Priorities are determined by Hillis, Spell, and Fridriksson together in monthly videoconferences via internet). Potential disputes are resolved by this core leadership.

###

### 10.3.3 Metrics

**Recruitment**

The Recruitment index (RI) which is the # of days required to recruit an analyzable participant at one site is a measure of quality control on recruitment. Our good historic retention rates seem to be due to our high quality, experienced speech-language pathologists who work with participants to accommodate their ability to participate throughout the course of the studies. These accommodations include excellent communication with participants and family members, the provision of lodging for out of town participants and reimbursement for mileage for those participants who drive long distances to participate.

**Assessment**

Measurements for assessment fidelity include rater reliability on our naming and discourse assessments (outcome measures), participant test-retest reliability on the naming assessment, how well SLPs adhere to assessment protocols and engage participants, and inter-rater reliability in evaluating severity of leukoaraiosis.

**Treatment**

Treatment sessions also are examined for adherence. A trained independent rater will watch videos of recorded sessions and rate the following: adherence to treatment procedures, frequency of additional, non-prescribed elements; frequency of omitted prescribed elements; frequency of cross-contamination; and clinician enactment and engagement.

### 10.3.4 Protocol Deviations

Protocol deviations will be recorded by any study team member who notices the deviation, and these will be reported to the IRB at the time of the annual review, unless the deviation caused potential harm or withdrawal of the participant from the study. In the latter case, the deviation will be reported to the IRB immediately

### 10.3.5 Monitoring

**Recruitment**

To increase the efficiency of recruitment and to ensure resources are not wasted due to participant withdrawal from the project, the Clinical Core monitors the rate of recruitment with a recently developed “recruitment index”. The Clinical Core has an adaptive plan for recruitment in place so that advertising and recruitment efforts are modified if the recruitment index shows the project is not on target for meeting proposed recruitment numbers.

**Assessment**

SLP assessment delivery is measured through direct observation or video recorded assessment sessions. A trained, independent rater watches videos of recorded sessions and rates the following for all assessments except the repeated measures (discourse, PNT): adherence to assessment administration procedures; frequency of additional, non-prescribed elements; frequency of omitted prescribed elements; frequency of cross-contamination; and assessor enactment and engagement. The rater conducts the implementation assessment for the repeated measures, as they already spend ample time observing the videos (as discourse transcription and PNT scoring often requires multiple viewings) and also have expert knowledge of the assessment procedures and scoring. Each assessor has 10% of their total assessment session time monitored for accurate implementation**.** Continuous monitoring of the delivery of the assessment will further guard against drift and contamination and alert the Clinical Core to the need for action if fidelity is compromised.

**Treatment**

Treatment fidelity has been carefully monitored through face-to-face and video recorded treatment sessions. This has been extremely helpful to make sure that all participants are receiving treatment in the same way. Dr. Spell observes one treatment session for each therapy type (e.g. one phonological session, one semantic session, one tDCS session) and evaluates both treatment delivery and treatment receipt for each participant. A treatment fidelity log is kept for each SLP. Each clinician will have 20% of their total treatment session time monitored quarterly for accurate implementation**.**

# 11. PARTICIPANT RIGHTS AND CONFIDENTIALITY

## 11.1 Institutional Review Board (IRB) Review

This protocol and the informed consent document and any subsequent modifications will be reviewed and approved by the IRB or ethics committee responsible for oversight of the study. The consent form should be separate from the protocol document.

## 11.2 Informed Consent Forms

A signed consent form will be obtained from each participant. For participants who cannot consent for themselves, such as those with a legal guardian (e.g., person with power of attorney), this individual must sign the consent form. The consent form will describe the purpose of the study, the procedures to be followed, and the risks and benefits of participation. A copy will be given to each participant or legal guardian and this fact will be documented in the participant’s record.

## 11.3 Participant Confidentiality

Participation in this study should not put participants in any legal risk, even in the case of a breach of confidentiality. We will undertake every effort to keep the information in the study confidential. Participants will be assigned a code number in order to keep the information confidential. The networks on which the information will be stored are password protected. Everybody involved in the study will have completed the appropriate HIPAA training and are fully aware of confidentiality issues. No names will be included in any publications resulting from this work

Any data, specimens, forms, reports, video recordings, and other records that leave the site will be identified only by a participant identification number (Participant ID, PID) to maintain confidentiality. All records will be kept in a locked file cabinet. All computer entry and networking programs will be done using PIDs only. Information will not be released without written permission of the participant, except as necessary for monitoring by IRB, the FDA, the NIDCD, and the OHRP.

## 11.4 Study Discontinuation

The study may be discontinued at any time by the IRB, the NIDCD, the OHRP, the FDA, or other government agencies as part of their duties to ensure that research participants are protected. The study may be discontinued if the study is closed by Johns Hopkins School of Medicine or C-STAR.

# 12. COMMITTEES

None.

# 13. PUBLICATION OF RESEARCH FINDINGS

Publication of the results of this trial will be governed by the policies and procedures developed by the Center for the Study of Aphasia Recovery (C-STAR).

# 14. REFERENCES

Bhogal SK, Teasell R, Speechley M. Intensity of aphasia therapy, impact on recovery. Stroke. 2003;34(4):987-993.

Brady MC, Kelly H, Godwin J, Enderby P. Speech and language therapy for aphasia following stroke. The Cochrane Library. 2012.

Chollet F, Tardy J, Albucher J, et al. Fluoxetine for motor recovery after acute ischaemic stroke (FLAME): A randomised placebo-controlled trial. The Lancet Neurology. 2011;10(2):123-130.

Dennis M, Mead G, Forbes J, Graham C, Hackett M, Hankey GJ, House A, Lewis S, Lundström E, Sandercock P, Innes K. Effects of fluoxetine on functional outcomes after acute stroke (FOCUS): a pragmatic, double-blind, randomised, controlled trial. The Lancet. 2018 Dec 5.

Doron R, Lotan D, Versano Z, et al. Escitalopram or novel herbal mixture treatments during or following exposure to stress reduce anxiety-like behavior through corticosterone and BDNF modifications. PloS one. 2014;9(4):e91455.

Enderby P, Broeckx J, Hospers W, Schildermans F, Deberdt W. Effect of piracetam on recovery and rehabilitation after stroke: A double-blind, placebo-controlled study. Clin Neuropharmacol. 1994;17(4):320-331.

Fridriksson J, Elm J, Stark BC, et al. BDNF genotype and tDCS interaction in aphasia treatment. Brain stimulation. 2018. (e-pub ahead of print)

Gu SC, Wang CD. Early selective serotonin reuptake inhibitors for recovery after stroke: a meta-analysis and trial sequential analysis. Journal of Stroke and Cerebrovascular Diseases. 2018 May 1;27(5):1178-89.

Hayasaka Y, Purgato M, Magni LR, et al. Dose equivalents of antidepressants: Evidence-based recommendations from randomized controlled trials. J Affect Disord. 2015;180:179-184.

Hilari K, Byng S, Lamping DL, Smith SC. Stroke and aphasia quality of life scale-39 (SAQOL-39) evaluation of acceptability, reliability, and validity. Stroke. 2003; 34(8): 1944-50.

Hillis AE. The 'standard' for poststroke aphasia recovery. Stroke 2010; 41: 1316-1317.

Hillis AE. Influence of selective serotonin reuptake inhibitors on aphasia recovery:

Preliminary data from a clinical trial. To be presented at the International Stroke Conference, Honolulu, Hawaii, Feb 2019. Abstract in Stroke, in press.

Hillis AE, Beh YY, Sebastian R, et al. Predicting recovery in acute poststroke aphasia. Ann Neurol. 2018;83(3):612-622.

Hillis AE, Tippett DC. Stroke recovery: Surprising influences and residual consequences. Advances in Medicine. 2014;2014.

Huber W, Willmes K, Poeck K, Van Vleymen B, Deberdt W. Piracetam as an adjuvant to language therapy for aphasia: A randomized double-blind placebo-controlled pilot study. Arch Phys Med Rehabil. 1997;78(3):245-250.

Jorge RE, Acion L, Moser D, Adams HP, Robinson RG. Escitalopram and enhancement of cognitive recovery following stroke. Arch Gen Psychiatry. 2010;67(2):187-196.

Kraglund KL, Mortensen JK, Damsbo AG, Modrau B, Simonsen SA, Iversen HK, Madsen M, Grove EL, Johnsen SP, Andersen G. Neuroregeneration and Vascular Protection by Citalopram in Acute Ischemic Stroke (TALOS) A Randomized Controlled Study. Stroke. 2018 Nov;49(11):2568-76.

Kurland J, Pulvermuller F, Silva N, Burke K, Andrianopoulos M. Constrained versus unconstrained intensive language therapy in two individuals with chronic, moderate-to-severe aphasia and apraxia of speech: Behavioral and fMRI outcomes. American journal of speech-language pathology. 2012;21(2):S87.

Lam RW. Psychopharmacology for the clinician. Journal of psychiatry & neuroscience: JPN. 2013;38(2):E5.

Lazar RM, Minzer B, Antoniello D, Festa JR, Krakauer JW, Marshall RS. Improvement in aphasia scores after stroke is well predicted by initial severity. Stroke. 2010;41(7):1485-1488.

Marangolo P, Fiori V, Sabatini U, et al. Bilateral transcranial direct current stimulation language treatment enhances functional connectivity in the left hemisphere: Preliminary data from aphasia. J Cogn Neurosci. 2016;28(5):724-738.

Mead GE, Hsieh CF, Lee R, Kutlubaev MA, Claxton A, Hankey GJ, Hackett ML. Selective serotonin reuptake inhibitors (SSRIs) for stroke recovery. Cochrane Database of Systematic Reviews. 2012(11).

Pan XL, Chen HF, Cheng X, Hu CC, Wang JW, Fu YM, Kong HM, Shao HJ. Effects of Paroxetine on Motor and Cognitive Function Recovery in Patients with Non-Depressed Ischemic Stroke: An Open Randomized Controlled Study. Brain Impairment. 2018 May:1-7.

Sæterdal I, Pike E, Ringerike T, Gjertsen MK. Efficacy and safety for the newer antidepressants in adults. . 2007.

Sanchez C, Reines EH, Montgomery SA. A comparative review of escitalopram, paroxetine, and sertraline: Are they all alike? Int Clin Psychopharmacol. 2014;29(4):185.

Sebastian R, Saxena S, Tsapkini K, et al. Cerebellar tDCS: A novel approach to augment language treatment post-stroke. Frontiers in human neuroscience. 2017;10:695.

Sinclair VG, Wallston KA. The development and psychometric evaluation of the Brief Resilient Coping Scale. Assessment. 2004;11(1).

Soto CJ & John OP. The next Big Five Inventory (BFI-2): Developing and assessing a hierarchical model with 15 facets to enhance bandwidth, fidelity, and predictive power. Journal of Personality and Social Psychology. 2017;113(1): 117.

Spitzer RL, Krownke K, Williams JB, Löwe B. A brief measure for assessing generalized anxiety disorder: the GAD-7. Archives of Internal Medicine. 2006;166(10):1092-1097.

Wang C, Zhang Y, Liu B, Long H, Yu C, Jiang T. Dosage effects of BDNF Val66Met polymorphism on cortical surface area and functional connectivity. Journal of Neuroscience. 2014;34(7):2645-2651.

Walker-Batson D, Curtis S, Natarajan R, et al. A double-blind, placebo-controlled study of the use of amphetamine in the treatment of aphasia. Stroke. 2001;32(9):2093-2098.

Wang C, Zhang Y, Liu B, Long H, Yu C, Jiang T. Dosage effects of BDNF Val66Met polymorphism on cortical surface area and functional connectivity. Journal of Neuroscience. 2014;34(7):2645-2651.

Watson D, Clark LA, Tellegen A. Development and validation of brief measures of positive and negative affect: the PANAS scales. Journal of personality and social psychology. 1988; 54(6): 1063.

Weathers FW, Blake DD, Schnurr PP et al. The Life Events Checklist for DSM-5 (LEC-5). 2013. Instrument available from the National Center for PTSD at www.ptsd.va.gov

Zimet GD, Dahlem NW, Zimet SG, Farley GK. The multidimensional scale of perceived social support. Journal of Personality Assessment. 1988; 52(1):30-41.

# 15. SUPPLEMENTS/APPENDICES

Appendix A: Informed consent form

Appendix B: Suicidality Risk Minimization Plan

**Suicidality Risk Minimization Plan**

Escitalopram and all other drugs in its class (selective serotonin reuptake inhibitors) have a “black box warning” in the label that reads in relevant part: “Increased risk of suicidal thinking and behavior in children, adolescents and young adults taking antidepressants for major depressive disorder (MDD) and other psychiatric disorders.” There has been a longstanding concern that antidepressants may have a role in inducing worsening of depression and the emergence of suicidality in patients 18-24 with MDD during the early phases of treatment. Short-term studies did not show an increase in the risk of suicidality with antidepressants compared to placebo in adults beyond age 24 and **show a reduction in suicidality with antidepressants compared to placebo in adults aged 65 and older.**

**Participants whose Patient Health Questionnaire (PHQ-9) affective screening tool performance indicates moderately severe depression (>15) or who endorse suicidal ideation (either on PHQ-9 question 9 or in general) will be excluded from the study or, if already enrolled, will be withdrawn from the study, and referred for psychiatric evaluation.**

In recognition of the prevalence of post-stroke depression (20-60%), the language difficulties anticipated in our patient sample, and the difficulties in assessing true suicidality risk without the ability to conduct a gold-standard psychiatric clinical interview or administer the Columbia Suicide Severity Rating Scale (C-SSRS), and out of an abundance of caution, we have built a constellation of criteria across additional affective measures that, if satisfied, will result in a referral to the site psychiatrist who will make the final determination about the appropriate course of action regarding continuation of study participation.

The following criteria result in **exclusion or immediate withdrawal** from the study and **informed referral to the site psychiatrist**:

- PHQ-9 > 15
- PHQ-9 question 9 endorsement (i.e., any response other than “never”)

If a participant endorses suicidal ideation, the following follow-up questions should be administered immediately and documented on the PHQ-9:

1. Are you having thoughts of suicide?
2. Do you have a plan for completing suicide?
3. Do you have access to what you would use to kill yourself?
4. Do you have a time planned to take your life?

**If all four questions are endorsed “Yes,” IMMEDIATELY:**

**Inpatient: Notify the site PI and the nurse on duty**

**Outpatient/Home: Notify any family/caregiver present and call 9-1-1**

If any question is endorsed “Yes,” include this information in the informed referral to the site psychiatrist.

The following criteria result in **informed referral** to the site psychiatrist, who will make the final determination regarding withdrawal from the study:

- Any lifetime history of suicidal ideation or attempts
- Any lifetime history of MDD or other previous psychiatric diagnosis and treatment
- Mean < 4 on Multidimensional Scale of Perceived Social Support at baseline

AND

- Week-by-week increase of 5 or more points on PHQ-9 administered during weekly monitoring phone calls

**Informed referrals should include**:

Brief introduction of yourself and purpose for email

Participant’s name, age, and MRN

Duration of time on escitalopram and pill counts/statement regarding presumed compliance

All information regarding suicidal ideation collected to date:

- Medical history
- Social history
- All PHQ-9 administrations
- Multidimensional Scale of Perceived Social Support
- Stroke Aphasia Quality of Life Scale-39
- Positive and Negative Affect Schedule-SF
- Life Events Checkist-5
- Big Five Inventory 2 Ne items
- General Anxiety Disorder-7
- Brief Resilient Coping Scale

This is particularly important as the common tools for assessing suicidality tend to place heavy linguistic demands that may not be appropriate for many of our participants.

**Please err on the side of caution with all participants and refer any additional questions to the site PI.**

**Further reading:**

Escitalopram oxalate (Lexapro) label, including suicidality risk assessment by age: <https://www.accessdata.fda.gov/drugsatfda_docs/label/2017/021323s047lbl.pdf>

Post-stroke depression review:

Robinson, R. G., & Jorge, R. E. (2016). Post-stroke depression: a review. *American Journal of Psychiatry*, *173*(3), 221-231.
