# Supplementary material for: Protocol for Escitalopram and Language Intervention for Subacute Aphasia (ELISA): A randomized, double blind, placebo-controlled trial
Source: PLoS One. 2021 Dec 23;16(12):e0261474. doi: 10.1371/journal.pone.0261474 (PMC8699636; doi:10.1371/journal.pone.0261474)
Supplement: S3 File — (PDF) [file pone.0261474.s003.pdf]

INFORMED CONSENT FORM

Title of Project: **Escitalopram and Language Intervention for Subacute Aphasia (ELISA)**

Principal Investigator: **Argye Hillis-Trupe, M.D.**
Phipps 446, 600 N. Wolfe Street,
Baltimore MD 21287
Phone (410) 812-6716
Fax (410) 614-9807

Participant's Printed Name: ____

INTRODUCTION

You are being asked to take part in a research study (Escitalopram and Language Intervention for Subacute Aphasia; ELISA), which seeks to identify a more effective means of treating post-stroke language difficulties. This is a multi-site study, meaning it will take place at several different locations. Because this is a multi-site study, this informed consent form includes two parts. The first part of this document includes information that applies to all study sites. The second part of the consent form includes information specific to the study site where you are being asked to enroll.

Participation in this study is voluntary. Even if you decide to join now, you can change your mind later. The person being asked to be in this research study may not be able to give consent to be in this study. You are therefore being asked to give permission for this person to be in the study as his/her decision maker. We urge you to discuss any questions about this study with our staff members. Talk to your family and friends about it and take your time to make your decision. If you decide to participate, you must sign this form to show that you want to take part.

Research Summary (Key Information):

The information in this section is intended to be an introduction to the study only. Complete details of the study are listed in the sections below. If you are considering participation in the study, the entire document should be discussed with you before you make your final decision. You can ask questions about the study now and at any time in the future.

This research is being done to see if a study drug called escitalopram may improve language therapy effectiveness in individuals with aphasia (language difficulties) within 3 months after a stroke. You will be in the main part of the study for about 1 year including follow-up testing, depending on your availability. First you will have an initial screening, which may include a picture of your brain called a magnetic resonance image (MRI) or a series of measurements taken using near-infrared light (NIRS). You can still participate in the study if you do not want to have the MRI or NIRS, and you may not be offered one or both of these studies. Next, you will either be assigned to receive escitalopram or a placebo (an inactive material that does not contain any active study drug), to be taken daily for 3 months (90 days). Starting 2 months after your stroke, if you still have language problems caused by the stroke, you will receive 15 sessions of naming treatment over the course of 3-4 weeks. The treatment is delivered on a computer and takes 45 minutes each session.

Section 1. PURPOSE OF THE RESEARCH

You are being asked to take part in this research study because you have recently experienced a stroke. This research is being done to help us understand whether and how the use of a drug, called escitalopram (a selective serotonin reuptake inhibitor or SSRI), may improve language therapy effectiveness, as measured when naming untrained pictures and describing pictures, in individuals with aphasia within three months after a stroke.

Another goal of part of this study is to investigate the particular concentration of certain molecules in the brain of people with following a stroke and how the SSRI might change this. This will allow us to better understand how brains change in people receiving language therapy. Not everyone in the study will participate in the MRI or NIRS part of the study. You may elect not to participate in the MRI part, or you may not be able to participate in that part. You also may elect not to participate in the NIRS part.

Are there any investigational drugs/devices/procedures?

Escitalopram is approved by the Food and Drug Administration (FDA) for the treatment of major depressive disorder and generalized anxiety disorder. It is not approved for use in improving outcomes following stroke, and its use in this study is considered investigational.

Who can join this study?

People with language difficulties, called aphasia, following stroke may join, unless they are currently experiencing severe depression or suicidal ideation. You may not be permitted to join the study if you take certain drugs, as it may not be safe for you to take escitalopram at the same time.

How many people will be in this study?

We expect to enroll 88 people in the study.

Section 2. PROCEDURES

If you agree to be in this study, we will ask you to do the following things:

Screening (Visit 1)

If you agree to be in this study, we will ask you to complete some of the tests and procedures to see if you can be in the study:

- You will receive a neurological examination and your medical history will be considered.
- Your heart will be examined again using an electrocardiogram. This quick tool allows us to measure the rhythm of your heart using wires attached to stickers placed on your chest.
- You will be asked for a blood sample. This is done to monitor your body's response to the study drug.
- If you are currently taking certain drugs, it may not be safe for you to take escitalopram at the same time. These drugs include monoamine oxidase inhibitors (MAOIs) and pimozide. You will be asked about your current medications, including whether you take over-the-counter drugs, vitamins, or supplements regularly. **Please alert the study doctor if you begin or discontinue any medication during the study.**

If you do not qualify, you will not participate further. If you do qualify, we will ask you to do the following things:

1 Week after Enrollment (Visit 2)

About 1 week after entering the study, we will first ask you some speech and language questions. You will be asked questions and given the opportunity to provide longer descriptions so we can assess your language in many different ways. Some of these assessments will be done on the computer. Others will be done in person.

You also will be asked for a saliva sample. This is being done to test for healthy differences in a gene called Brain Derived Neurotrophic Factor (BDNF) and FoxP2 that may impact your response to the study drug. The results do not have any known influence on your health. You may elect not give us a saliva sample and can still participate in the study

Please check box and sign to indicate your choice below:

YES ☐ ________________________
Signature of Participant

NO ☐ ________________________
Signature of Participant

During this visit, you also will be asked to have an MRI if it is safe for you to do so and if you elect to have it. MRI scans create images of the body using a strong magnet and radio waves. There is no radiation involved in an MRI exam. Most MRIs take about 60 minutes. You may not take part in this study if you have any metal or device in your body which is not compatible with MRI. Examples include certain pacemakers, defibrillators, aneurysm clips, or other implanted electronic or metallic devices, shrapnel, or other metal. If you have a history of metal in your head or eyes, you cannot take part in this study. The MRI machine periodically makes loud banging noises. We will provide earplugs or headphones for you to wear during the MRI exam. You may decline to have the MRI exam and still participate in the study.

Please check box and sign to indicate your choice below:

YES ☐ ________________________
Signature of Participant

NO ☐ ________________________
Signature of Participant

Whether or not you have an MRI, you may be offered and elect to have a different kind of imaging, called near-infrared spectroscopy (NIRS). NIRS measures brain activity using lights placed on the head by wearing a cap and takes about 60 minutes. It requires being in a darkened room while the image is acquired. This study may not be available at your location at the time of your enrollment.

Please check box and sign to indicate your choice below:

YES ☐ ________________________
Signature of Participant

NO ☐ ________________________
Signature of Participant

At the end of this visit you will be randomly (by chance, like a flip of a coin) assigned to receive either escitalopram or a placebo, to be taken daily for 3 months (90 days). A placebo is an inactive substance that looks like the study drug but contains no active drug. You and the study staff will not know if you are taking the escitalopram or the placebo, but this information can be made available in the case of an emergency.

For the first week of the study and last week of the study, you will take 5 mg (1 pill) of the study drug (escitalopram or placebo). This is done to minimize the discomfort that some people experience when starting or ending escitalopram. After the first week, the dose of the study drug will be increased to 10 mg (2 pills) each day

Visits 1 and 2 will typically occur during the acute hospitalization. The study drug (escitalopram or placebo) will be administered by the nursing staff while you are an inpatient (on the acute unit or inpatient rehabilitation unit).

Over the next three months (90 days), you and/or your caregivers will be called weekly to check that you are taking the pills that you have been assigned each day. These calls will allow you to discuss any bad effects or changes you may feel ("side effects"). The caller also will administer the Patient Health Questionnaire-9 (PHQ-9). The PHQ-9 helps us determine if you are currently experiencing severe depression. If you experience severe depression or suicidal ideation (thoughts of self harm or suicide), you will be removed from the study and referred to psychiatry for treatment.

For the first two months of your enrollment in the study, you will receive no language treatment as part of the study, but you will be expected to take the drug you have received each day, and you may have language therapy as part of your routine medical care.

Language Treatment (Visits 4-18)

Starting 2 months after your stroke, if you still have language problems, you will receive 15 sessions (Visits 4-18) of naming treatment over the course of 3-4 weeks. The treatment is delivered on a computer with the assistance of a staff member and takes 45 minutes. The 15 sessions of language treatment we provide will be provided in addition to any ongoing speech and language treatment.

Several times during your participation in the study, your language abilities will be tested using several measures. You will be asked to complete the language assessments, the PHQ-9 (assessment of depression) even if you do not have the language treatment. These assessments will allow us to determine the effects of the study drug. You will also receive neurological examination every week during this part of the study.

Follow-up (Visits 19- 21)

Visits 19 will be done about one week after language treatment. Visit 20 will be done about 5 weeks after the language treatment. Visit 21 will be done about 20 weeks after language treatment. During each of the follow-up visits, your language abilities will again be tested using several measures. If you were offered and agreed to the MRI test done during Visit 2, we will ask you to have one done again during Visit 19. If you were offered and agreed to the NIRS done during Visit 2, we will ask you to have one done again during Visit 19.

Photographs/Video recordings:

As part of this research, we are requesting your permission to create and use audio-visual recordings to help answer the research question. Any audio-visual recordings will not be used for advertising or non-study related purposes.

You should know that:

- You may request that the audio-visual recordings be stopped at any time.

- If you agree to allow the audio-visual recordings and then change your mind, you may ask us to destroy that imaging/recording. If the imaging/recording has had all identifiers removed, we may not be able to do this.

- We will only use these audio-visual recordings for the purposes of this research.

Please indicate your decision below by checking the appropriate statement:

______I **agree** to allow the study team members to make and use photographs/video recordings/audio recordings of me (or the participant I represent) for the purpose of this study.

______I **do not agree** to allow study team members to make and use photographs/video recordings/audio recordings of me (or the participant I represent) for the purpose of this study.

____ _______________
Participant Signature Date
(or Parent/Legally Authorized Representative Signature, if applicable)

Will research test results be shared with you?

If you request, we will share the results of the language and behavioral assessments and MRI with you, your doctors, or your speech-language pathologists. We will not share the results of the genetic tests (from the saliva sample) as we may not have that information until long after your participation in the study.

Section 3. TIME DURATON OF THE PROCEDURES AND STUDY

If you agree to take part in this study, you will be in the main part of the study for about 1 year including follow-up testing, depending on your availability. You will be asked to meet with members of the research team approximately 21 times. Evaluation sessions typically involve 6-8 hours of assessment, usually divided into 2-3 days. Treatment sessions take approximately 45-60 minutes.

Section 4. DATA AND BIOSPECIMENS

If you join this study, your data and biospecimens will be used to answer the research question and your data will be used to publish the findings of this study. Biospecimens may include any of the following: blood, tissue, saliva, urine, bone marrow, cells, etc. Most biospecimens contain DNA, which is the genetic code for each person.

You will not own the data and/or biospecimens collected from you as part of this research study. If researchers use them to create a new product or idea, including those that may have commercial value, you will not benefit financially from those efforts.

This study involves genetic testing on samples that you provide. The Genetic Information Nondiscrimination Act (GINA) is a federal law that helps reduce the risk of discrimination by health insurers or employers based on your genetic information. GINA does not protect you from discrimination if you apply for other types of insurance (such as life, disability or long-term care). GINA also does not protect you against discrimination based on an already-diagnosed genetic condition or disease.

Genetic information is unique to you and your family. Even without your name or other personal identifiers, it may be possible to identify you or other members of your family with your genetic information.

Johns Hopkins follows procedures so that people who work with your DNA information for research cannot discover it belongs to you, unless you have given consent. However, new techniques will likely make it easier to link your genetic data to you in the future, so we cannot promise that your genetic information will never be linked to you.

Johns Hopkins researchers and their collaborators may use the data/biospecimens collected in this study for future research purposes and may share some of the data/biospecimens with others.

Future research may include:

- Genetic research: Study of human DNA to find out what genes and environmental factors contribute to diseases. Each cell contains your complete DNA.
- Gene sequencing: Gene sequencing of your DNA provides researchers with the code to your genetic material

Because science constantly advances, we do not yet know what future use of research data or biospecimens may include. This future research may be unrelated to the current study and may include outside collaborators.

Sharing data and/or biospecimens is part of research and may increase what we can learn from this study. Often, data/biospecimen sharing is required as a condition of funding or for publishing study results. It also is needed to allow other researchers to validate study findings and to come up with new ideas. Your data and/or biospecimens may be shared with researchers at Johns Hopkins and other institutions, for-profit companies, sponsors, government agencies, and

other research partners. Your data and/or biospecimens may also be put in government or other databases/repositories.

We will do our best to protect and maintain your data/biospecimens in a safe way. One of the ways we protect data/biospecimens is by limiting the uses of the information and the type of information that is shared, especially your personal information. This may occur through data/specimen sharing agreements and review by oversight groups within Johns Hopkins.

If data/biospecimens are used or shared with types of information that may be likely to identify, you such as your name, address or medical record number, further institutional review and approval would be required. In these cases, Johns Hopkins will review whether additional consent from you is required.

Generally, if your data/biospecimens are used/shared without any personal identifiers or with information that is less likely to identify you (such as the date of a procedure), further review and approval is not needed.

Data/biospecimen sharing could change over time and may continue after the study ends. The use and sharing of your data and biospecimens is required for participation in this research study. If you are not comfortable with the use and sharing of your data/biospecimens in future research without further consent, you should not participate in this study.

Section 5. DISCOMFORTS AND RISKS

Escitalopram

Antidepressants, such as escitalopram, increased the risk of suicidal thinking and behavior (suicidality) in children, adolescents, and young adults in short-term studies of major depressive disorder (MDD) and other psychiatric disorders. However, there was a reduction in risk with antidepressants compared to placebo in adults aged 65 and older. Participants with anti-depressant use at the time of stroke onset or moderately severe depression (PHQ-9 score >15) at enrollment are excluded from the present study to minimize the possibility that individuals with MDD will be enrolled in the study.

The most common side effects of escitalopram are drowsiness, nausea, insomnia, dry mouth, constipation, which are also common with placebo.

An uncommon but more serious side effect is a change in heart behavior (QT prolongation), detectable on the heart evaluation (electrocardiogram). This side effect is being monitored through the regular use of heart evaluations throughout the study.

Escitalopram has been associated with spontaneous reports of adverse events occurring when people stop taking it, particularly when they do so abruptly, including the following:

- dysphoric mood
- irritability
- agitation

- dizziness
- sensory disturbances (e.g., paresthesias such as electric shock sensations)
- anxiety
- confusion
- headache
- lethargy
- emotional lability
- insomnia
- hypomania

These effects are minimized by starting and ending dosing with a smaller amount of escitalopram, called tapering. Participants will receive half of the full dose of the study drug each day for the first week of the study, then transition to the full dose of the study drug for the remaining 90-day duration. When the study ends, participants will receive half of the full dose of the study drug each day for two weeks after the 90 days. Participants will be monitored through weekly calls in order to provide opportunities to discuss these effects if they occur.

Low sodium in blood (hyponatremia) can occur as a result of treatment with escitalopram, in many cases due to the development of the syndrome of inappropriate antidiuretic hormone secretion (SIADH). Elderly patients may be at greater risk of developing hyponatremia with SSRIs. Also, patients taking diuretics or who are otherwise volume depleted may be at greater risk. Signs and symptoms of hyponatremia include headache, difficulty concentrating, memory impairment, confusion, weakness, and unsteadiness, which may lead to falls. We will monitor sodium using periodic blood tests (electrolytes) and discontinue treatment in people who develop serious hyponatremia (Na < 130).

If you are in the treatment group that receives placebo (inactive substance) your language difficulties may not improve.

Blood Draw

Taking blood may cause discomfort, bleeding, or bruising where the needle enters the body. In rare cases, it may result in fainting. There is a small risk of infection.

MRI

While no significant risks have been found from the use of MRI scans, you may be bothered by the noise made by the MRI scanner and by feelings of being closed in (claustrophobia).

fNIRS

While no significant risks have been found from the use of NIRS, you may feel uncomfortable in dark rooms (scotophobia), such as those required in fNIRS.

Questions/Language Evaluation

You may get tired or bored when we are asking you questions, or you are completing language evaluation. You do not have to answer any question you do not want to answer. If language and cognitive evaluation makes you feel extremely uncomfortable and distressed, the

examiner may ask you to take a break. If this happens constantly and you get constantly frustrated so that you cannot tolerate assessment, the examiner may ask you to quit the study without any consequences to your regular medical care.

Identifiable Private Information

There is the risk that information about you may become known to people outside this study.

Unknown Risk

There may be side effects and discomforts that are not yet known.

Are there risks related to pregnancy?

You will not be eligible to participate in the study if you are pregnant, as the effects of escitalopram on the fetus are not well known. If you become pregnant or intend to become pregnant during the study, please notify the study doctor. This research may hurt an embryo or fetus in ways we do not currently know.

Section 6. POTENTIAL BENEFITS

There may or may not be a direct benefit to you from being in this study. Your language function might improve with language therapy or with study drug, but we do not know yet if it will improve. If you participate in this study, it may help others in the future.

Section 7. STATEMENT OF CONFIDENTIALITY

7.1 Privacy and Confidentiality Measures

We will undertake every effort to keep the information in the study confidential. Participants will be assigned a code number in order to keep the information confidential. The networks on which the information will be stored are password protected. Everybody involved in the study will have completed the appropriate HIPAA training and are fully aware of confidentiality issues. No names will be included in any publications resulting from this work.

7.2 The Use of Private Health Information

Health information about you will be collected if you choose to be part of this research study. Health information is protected by law, as explained in the site-specific information part of this consent form.

Representatives of the following people or groups may use your health information and share it with other specific groups in connection with this research study.

- The principal investigator, Dr. Argye Hillis-Trupe
- Other investigators across study sites
- The study coordinators and research teams across study sites
- The Institutional Review Board of any study site
- The Human Subjects Protection Office of any study site
- The pharmacy of any study site

During the study, we will tell you if we learn any new information that might affect whether you wish to continue to participate.

A description of this clinical trial will be available on http://www.ClinicalTrials.gov, as required by U.S. Law. This Web site will not include information that can identify you. At most, the Web site will include a summary of the results. You can search this Web site at any time. If you would like to review the information for this study, or a summary of the results, ask the study team doctor for the ClinicalTrials.gov study registration number.

During this study, you will not have access to certain medical information and test results collected for study purposes. If an emergency occurs while you are in the study, medical information needed for your treatment can be made available to your study doctor and other physicians who treat you. When the study is completed, all the information in your medical record will be available to you.

Section 8. COSTS FOR PARTICIPATION

Details of cost for participation are specific to each study site.

Please refer to the **Site-Specific Consent Information (SSCI)**.

Section 9. COMPENSATION FOR PARTICIPATION

Details of compensation for participation are specific to each study site.

Please refer to the **Site-Specific Consent Information (SSCI)**.

Section 10. RESEARCH FUNDING

This work is supported by a grant from the National Institute on Deafness and Other Communication Disorders (NIH/NIDCD): P50 DC011739.

Section 11. CERTIFICATE OF CONFIDENTIALITY

Your study information is protected by a Certificate of Confidentiality. This Certificate allows us, in some cases, to refuse to give out your information even if requested using legal means.

It does not protect information that we have to report by law, such as child abuse or some infectious diseases. The Certificate does not prevent us from disclosing your information if we learn of possible harm to yourself or others, or if you need medical help.

Disclosures that you consent to in this document are not protected. This includes putting research data in the medical record or sharing research data for this study or future research. Disclosures that you make yourself are also not protected.

Section 12. VOLUNTARY PARTICIPATION

Taking part in this research study is voluntary. If you choose to take part in this research, your major responsibilities will include taking the study drug as prescribed and participating in treatment and evaluation activities. You do not have to participate in this research. If you choose to take part, you have the right to stop at any time. If you decide not to participate or if you decide to stop taking part in the research at a later date, there will be no penalty or loss of benefits to which you are otherwise entitled.

Your investigator may take you out of the research study without your permission. Some possible reasons for this are:

- You do not follow study procedures
- You become pregnant during the study
- You experience severe depression or other psychiatric or neurological condition, including any condition that would require a treatment unable to be taken with the study drug
- You exhibit medical characteristics that would make it unsafe for you to continue the use of the study drug (e.g., hyponatremia, QTc prolongation)

Also, NIH may end the research study early. If your participation in the research ends early, you may be asked to visit the investigator for a final visit.

If you will be participating in another clinical trial while in this research, you should discuss the procedures and/or treatments with your physician or the investigators. This precaution is intended to protect you from possible side effects from interactions of research drugs, treatments, or testing.

Section 13. CONTACT INFORMATION FOR QUESTIONS OR CONCERNS

You have the right to ask any questions you may have about this research.

If you have questions, complaints, or concerns or believe you may have developed an injury related to this research, please refer to the **Site-Specific Consent Information (SSCI) Form** for contact information at your enrollment site. You may also contact the lead Principal Investigator, Dr. Argye Hillis-Trupe at 410-812-6716. If you wish, you may contact the principal investigator by letter. The address is on page one of this consent form.

What is the Institutional Review Board (IRB) and how does it protect you?

This study has been reviewed by an Institutional Review Board (IRB), a group of people that reviews human research studies. The IRB can help you if you have questions about your rights as a research participant or if you have other questions, concerns or complaints about this research study.

For this multi-site study, Johns Hopkins has agreed to serve as the single IRB (sIRB) providing oversight for all sites. You may contact the Johns Hopkins IRB at 410-502-2092 or jhmeirb@jhmi.edu with your questions or concerns.
